# Engineered exosomes and composite biomaterials for tissue regeneration

**DOI:** 10.7150/thno.93088

**Published:** 2024-03-03

**Authors:** Weikang Hu, Wang Wang, Zesheng Chen, Yun Chen, Zijian Wang

**Affiliations:** 1 Department of Urology, Cancer Precision Diagnosis and Treatment and Translational Medicine Hubei Engineering Research Center, Zhongnan Hospital of Wuhan University, Wuhan 430071, China.; 2 Ministry of Education Key Laboratory of the Green Preparation and Application for Functional Materials, Hubei Key Laboratory of Polymer Materials, School of Materials Science and Engineering, Hubei University, Wuhan 430062, China.; 3 Department of Biomedical Engineering, Hubei Province Key Laboratory of Allergy and Immune Related Disease, TaiKang Medical School (School of Basic Medical Sciences), Wuhan University, Wuhan 430071, China.

**Keywords:** Exosomes, Tissue Engineering, Extracellular Vesicles, Stem Cell Therapy, Genetic Regulation

## Abstract

Exosomes, which are small vesicles enclosed by a lipid bilayer and released by many cell types, are widely dispersed and have garnered increased attention in the field of regenerative medicine due to their ability to serve as indicators of diseases and agents with therapeutic potential. Exosomes play a crucial role in mediating intercellular communication through the transfer of many biomolecules, including proteins, lipids, RNA, and other molecular constituents, between cells. The targeted transport of proteins and nucleic acids to specific cells has the potential to enhance or impair specific biological functions. Exosomes have many applications, and they can be used alone or in combination with other therapeutic approaches. The examination of the unique attributes and many functions of these factors has emerged as a prominent field of study in the realm of biomedical research. This manuscript summarizes the origins and properties of exosomes, including their structural, biological, physical, and chemical aspects. This paper offers a complete examination of recent progress in tissue repair and regenerative medicine, emphasizing the possible implications of these methods in forthcoming tissue regeneration attempts.

## 1. Introduction

### 1.1 The origin, biological function and isolation of exosomes

Exosomes, which are also known as natural lipid nanoparticles, are a subset of extracellular vesicles released from cells under physiological conditions or specific pathological circumstances 1. Exosomes are nanovesicles ranging from 30 to 150 nm in length, have a characteristic cup-shaped morphology, and are produced by endocytosis of the cytoplasmic membrane 2-4. Exosomes form within cells by the inward budding of late endosomes/multivesicular bodies. Exosomes are released extracellularly when these multivesicular bodies fuse with the plasma membrane 5. Exosomes were once thought to be a type of cellular metabolic waste. However, it has now been shown that these factors play a variety of crucial roles 6,7.

Exosomes can be found in bodily fluids, such as blood and urine, and are be transported to target cells at distant sites, similar to the conventional endocrine process 8. After being released, exosomes can act on specific target cells in proximity to the parent cell in a paracrine manner. Because the biomolecules in exosomes may vary in amount or composition after the development of certain diseases or injuries, they may be used as biomarkers of disease diagnosis, prognosis, and even damage 9.

Exosome isolation and characterization are active areas of research to identify biomarkers and understand cell‒cell communication 10. Exosomes vary in size, composition, function, and source, which makes isolation challenging. Additionally, most separation techniques cannot fully distinguish exosomes from extracellular vesicles originating from nonendosomal pathways or from lipoproteins with comparable physical properties. This results in low exosomal purity 11. Various methods are chosen for different purposes and applications. However, ultracentrifugation, size-based isolation approaches, polymer precipitation, density gradient isolation, commercially available kits, and affinity capture techniques are the most frequently used methods to isolate exosomes based on specific biomarkers and particle sizes 12.

### 1.2 The structure and components of exosomes

Similar to liposomes, exosomes are composed of internal aqueous medium and a lipid membrane. However, research has shown that exosomes have a complex structure that includes many lipids and proteins 13. Exosomes are protected by a lipid bilayer that is rich in ceramide, sphingomyelin, and cholesterol 14. Some tetraspanins, including CD9, CD63, and CD81, which can be used as markers to detect exosomes, are also abundant in the exosome membrane 15. Exosomes contain an abundance of unique biomolecules, proteins, and nucleic acids, including DNA, miRNAs, and messenger RNA (mRNA) 16,17. Due to their lipid bilayer membrane structure, these factors are stable and can disseminate even in the challenging tumor microenvironment 18. As a result of the reduced immunogenicity and toxicity due to the bilayer lipid membrane, the regular entry of exosomes into the extracellular space is supported 19. Exosomes are highly biocompatible because they are endogenous 20. Exosomes also have improved cell and tissue penetration abilities. Exosomes can overcome the drawbacks of other drug delivery methods because of these qualities 21,22.

### 1.3 The use of exosomes

Exosomes have gained the attention of the scientific community due to their capacity to transfer information via proteins, nucleic acids, and other biomolecules. Exosomes are widely acknowledged for their capacity to facilitate intracellular communication through the transport of various biomolecules, including proteins and nucleic acids, which can facilitate their uptake by distant target cells through endocytosis 23,24. Therefore, exosomes can target a specific cell or many cells to enhance or interfere with specific biological processes 21. An increasing number of studies have shown that exosomes are vital mediators of paracrine communication and are widely biologically distributed 25. These extracellular vesicles have been found in almost all bodily fluids, including saliva, milk, amniotic fluid, serum, plasma, and urine 26. Exosomes are highly effective cellular delivery systems that can carry biologically active factors, such as proteins, lipids, and nucleic acids. Exosomes play a crucial role in the pathogenesis of diseases such as cancer, cardiovascular and neurodegenerative diseases, and viral infections and critical physiological processes 27. The exosome pathway of intercellular trafficking is essential for many functions and diseases, including immunity, tissue homeostasis, and regeneration. Exosomes support effective cell-to-cell communication and signaling across biological barriers, such as the blood‒brain barrier 12.

Exosomal functions have been extensively studied, and the possibility of controlling tissue repair and regeneration is becoming increasingly important; for example, exosomes could be an attractive alternative to numerous cell and tissue engineering techniques 28. For instance, several investigations of mesenchymal stem cell (MSC) transplantation for tissue regeneration have shown that MSCs primarily alter cells through paracrine signaling mediated by the exosomes they release 29,30. Thus, a cell-free approach that uses paracrine agents, such as exosomes, to encourage tissue repair and regeneration would avoid the potential risks of direct stem cell transplantation, including teratomas, immunological rejection, and the decreased capacity of the engrafted cells to mediate tissue regeneration 31,32.

MSC-derived exosomes are exciting candidates for cell-free therapeutics because of the crucial functions of MSCs and their products in tissue regeneration 33-36. In addition, exosomes produced by immune cells, including monocytes, leukocytes, granulocytes, and lymphocytes, are thought to play roles in several essential biological processes, including neovascularization, coagulation, and the recruitment of inflammatory cells 37. As a result, they can ensure an appropriate inflammatory response after injury, which facilitates tissue repair and regeneration 38-41.

Exosomes exhibit superior biocompatibility and reduced toxicity compared to synthetic nanomaterials. Additionally, exosomes can cross the blood‒brain barrier (BBB) 42,43. Exosomes possess specific characteristics that render them promising nanomaterials for use in tissue regeneration, drug delivery, and gene delivery 26,39,40,44-50.

## 2. The characterization of exosomes

### 2.1 Exosomes are structurally stable and bioactive

Exosomes are structurally stable and bioactive vesicles that are produced by most cell types and play essential roles in intercellular communication. These factors possess a lipid bilayer membrane and are composed of progenitor cell-derived proteins, mRNAs, and miRNAs. A compact lipid membrane encases the contents of exosomes, preventing their degradation. This facilitates their transport through bodily fluids and preserves their cargo, which can engage in interactions with recipient cells. Exosomes transport functional biomolecules, such as RNAs and proteins, which can modulate gene expression within target cells 51,52. Exosomes can affect many biological pathways and processes 53-55. Exosomes may play a significant role in cell-to-cell communication. Exosomes facilitate a variety of processes, including immune responses, antigen presentation, RNA interference, and tumor progression, by transferring bioactive molecules between cells 56,57. Due to the presence of molecular constituents that originate from the cells that produce exosomes, exosome contents may be diagnostic tools and biomarkers for a wide range of diseases, including cancer. The primary attributes of exosomes are the stability, functionality, and the ability to transport bioactive cargo, which enables cellular communication and affects both local and systemic biological processes. However, their clinical use and biology remain primary concerns in biomedical research 12,16,21,24.

### 2.2 Exosomes are rich in factors and require efficient purification

Natural sources of molecular cargo that are profuse and rich in exosomes can be purified for further research. Exosomes are present in cell culture media, blood, urine, saliva, breast milk, and the majority of bodily fluids 26. Efficient access to these biofluids is a characteristic that renders exosomes highly suitable for sample collection and biomedical analysis 58.

Exosomes are composed of a lipid bilayer membrane and surface proteins that differ between cell types but share a set of conserved proteins. Exosomes are typically smaller (between 15 and 75 nm) than other extracellular vesicles, necessitating unique separation and analysis methods 59. Simple methodologies such as ultracentrifugation, density gradients, immunoaffinity capture, and precipitation kits can be used to purify or isolate exosomes. Researchers are able to extract exosomes using these isolation methods to conduct subsequent omics profiling assays and characterization investigations 60,61. In contrast to invasive techniques such as tissue biopsy, purified exosomes offer a concentrated and enriched reservoir of biomarkers.

The use of omics technologies to analyze exosome-encapsulated metabolites, proteins, lipids, and nucleic acids has significantly increased since their development. Exosome contents can now be correlated with disease subtypes, treatment responses, clinical outcomes, and diagnosis by cataloging and mapping biomolecular cargo under various conditions 9.

Exosome purification presents several challenges, primarily due to the small size of exosomes, the complexity of the biological fluids they are found in, and the need for high purity and yield 62-64. Many types of exosome separation, purification, and analysis have been performed; however, to date, no methodology has been found that offers sufficient robustness in terms of yield, selectivity, and reproducibility 59. To achieve desired purification outcomes, a combination of techniques is typically required due to the inherent biochemical properties of these vesicles and their vast differences based on the matrix from which they are obtained. Various physical and chemical methodologies have been developed to optimize exosome purification and yields. Table 1 presents descriptions of the various exosome isolation techniques that are commonly used, as well as their recommended applications 59,65.

In summary, isolating high-quality, well-characterized exosome preparations is still a technically demanding endeavor that faces multiple challenges. Advancing purification and analytical technologies to overcome these hurdles is an area of active research. Typically, the choice of exosome isolation method should be based on the specific needs of the subsequent analysis. Combining different techniques can enhance the efficiency of isolation. However, it is important to take into account the additional time, reagents, and cost. Moreover, implementing additional separation procedures can increase the error rate and potentially decrease the amount of exosomes that can be recovered. Therefore, a potential solution involves combining isolation and analysis methods. Given that immunoaffinity techniques can be used to isolate and characterize substances through a simple process, it is anticipated that these avenues will hold importance in the coming years. Developing a universally applicable method for recovering and purifying exosomes remains challenging. However, the key lies in standardizing current protocols that integrate techniques to address specific issues. The future of exosome isolation will undoubtedly be focused on translational technology, necessitating the creation of reliable and efficient protocols to accommodate upcoming uses in various settings.

To summarize, the purification of extracellular vesicles continues to pose a challenge, as scalable processes are needed to generate the required quantities of exosomes for clinical use 66. Currently, it is challenging to determine a single strategy that consistently produces the most superior quality of isolated exosomes with the desired characteristics. Typically, a combination of various methodologies is needed to attain optimal outcomes. Ultracentrifugation is expected to be a highly used method for obtaining exosomes. Novel strategies using immunoaffinity and microdevices are emerging as promising alternatives for obtaining these crucial nanoparticles.

### 2.3 Exosomes can be functionally modified

Exosomes can be functionally modified due to their intrinsic ability to establish connections with target cells. Research has developed methods to engineer and load exosomes with specific cargoes to modify the behavior of cells for therapeutic purposes 41. Parent cell engineering is possible; for instance, transfecting exosome-producing cells permits the packaging of modified proteins and RNAs into exosomes 67. This method enables cargo modification in the absence of direct exosome manipulation 68.

Direct exosome modification is an additional strategy. Purified exosomes can be loaded with bioactive substances such as ligands, microRNAs, and drugs through electroporation, sonication, transfection reagents, or membrane permeabilization using lipids or polymers 69. Surface functionalization involves the attachment of targeting moieties to the surface membrane of exosomes, which guides the delivery of their payloads to diseased cells and ensures their selective capture 70,71. The capacity to manipulate the molecular cargo of exosomes and direct their trafficking opens up enticing avenues for targeted drug delivery, disease-specific genetic therapy, and immune therapy 72.

However, the technical challenges of achieving controllable loading efficiency and scalable manufacturing persist. In addition, safety profiling pertaining to the specific impacts of engineered exosomes on the behavior of target cells is needed. After these issues are resolved, exosomes have the potential to emerge as exceptionally well-suited vehicles for therapeutic agents targeting native disease pathways via synthetic biology techniques.

## 3. Fabrication of exosome-loaded biomaterials

### 3.1 Exosomes can be grafted onto the surface of biomaterials

The use of exosomes to modify the surface characteristics of biomaterials used in regenerative medicine and tissue engineering has generated considerable interest. The bioactive payload and lipid-rich membrane of exosomes can regulate the efficacy, integration, and biocompatibility of scaffolds and devices 73. Grafted exosomes contain specific miRNAs, proteins, and lipids that stimulate signaling that promotes cell survival, proliferation, and functional performance in the microenvironments of target tissues. Surface coatings composed of exosomes promote the migration and adhesion of specific therapeutic cell types to scaffolds, thereby facilitating regeneration 74. Similar to biological Trojan horses, exosomes derived from patients can camouflage synthetic implants to prevent immunogenic reactions to foreign substances. Exosomes containing proangiogenic factors promote the formation of blood vessels, thereby enhancing engraftment and transplantation at implant sites 75. Initial proof-of-concept investigations have established that the bioactivity of exosomes can be efficiently transferred to compatible biomaterials. Further developments in the field of material science are necessary to effectively bind or embed isolated exosomes. Ensuring the safety of uncontrolled exosomal signaling continues to be a significant area of research.

### 3.2 Exosomes can be encapsulated inside biomaterials

Three-dimensional scaffolds enable the direct encapsulation of exosomes into bulk material or a porous network throughout the fabrication process, as opposed to relying on surface embellishment 76,77. This not only maintains the bioactivity of exosomes but also permits controlled and sustained release at specific sites. Crosslinking hydrogel precursors containing exosomes result in their uniform dispersion, followed by progressive release via diffusion or enzymatic/hydrolytic degradation. Protected exosome payloads can be encapsulated within microspheres and nanoparticles composed of lipids, polymers, or organic compounds until environmental stimuli initiate cargo discharge. Exosome loading is facilitated by porous three-dimensional scaffolds, which also permit elution after scaffold integration within tissue defects. By providing protection and sustained release, encapsulation eliminates the drawbacks associated with the limited half-life and stability of isolated exosomes 78. Additionally, the scaffold serves as a template for support at the exact sites of damaged tissue. However, the customization of release kinetics, loading efficiency, and storage stability necessitate additional material engineering upgrades. In addition, functional measurements of exosome bioactivity in clinically relevant in vivo models are needed 79-82. Innovative strategies for biomaterial design and cell-free therapeutic methods reveal the possibilities of exosomes in the realm of tissue regeneration.

### 3.3 Exosomes can be integrated with nanoparticles

An emerging strategy is the integration of exosomes and nanoparticles, which aims to use the benefits of both components to deliver therapeutic cargoes more efficiently 83. By using surface conjugation and encapsulation techniques, these natural and synthetic nanoscale vehicles are being fused, but the research is in the early stages. Surface coatings and other nanoparticle characteristics can increase the stability of exosomes and prolong their bioavailability. The use of targeting ligands conjugated to nanoparticles affords exosomes active guidance capabilities. The encapsulation of exosome cargo within nanoparticles protects drugs and delicate therapeutics 41,79. By engineering nanoparticles to react with stimuli such as temperature, pH, or enzymatic activity, exosomes can be released into target tissues in response to a trigger.

The use of fluorescent, contrast, or magnetic agents permits in vivo monitoring of exosome migration to specific sites 22,72,84,85.

The use of nanoparticle modular engineering and multifunctional characteristics can surmount to the limitations of exosome delivery and bioactivity 86. Matching biophysical properties while preventing the disruption of natural exosome functions continues to be a technical challenge. In addition, further research will be needed to address scalability and safety concerns as hybrid systems progress through preclinical studies.

## 4. Applications of exosome-loaded biomaterials

### 4.1 Neural regeneration

Peripheral nerve injuries severely affect patients and society and mostly affect working-age individuals. Despite advances in care, patients often have significant functional disabilities 87. Exosomes are promising therapeutic candidates for neural regeneration by restoring and inducing proliferation in neural tissue in the central nervous system (CNS) after injury or disease 88. Exosomes are attractive candidates for neural regeneration due to their unique properties. Exosomes facilitate communication between neurons and glia and affect central nervous system functions. In response to toxic or pathological stimuli in the brain, glial exosomes containing inflammatory molecules can communicate with neurons and contribute to neuroinflammation and neurodegenerative disorders. Due to their small size, exosomes can cross the blood‒brain barrier and be used as biomarkers and diagnostic agents for brain disorders and neuropathologies 42.

Exosomes derived from neural stem cells (NSCs) and mesenchymal stem cells (MSCs) have been studied for their ability to regenerate the CNS 89. The culture media of these cells can be used to isolate exosomes that can be delivered to sites of neural injury or degeneration. Studies have shown that exosomes can promote neural regeneration through multiple mechanisms. They can enhance the survival and proliferation of neural progenitor cells, which generate new neurons and supporting cells in the CNS. Exosomes also help neural progenitor cells mature into neurons and integrate into neural circuits. Exosomes modulate the immune response and reduce CNS inflammation, promoting neural regeneration. Furthermore, exosomes contain various bioactive molecules, including growth factors, neurotrophic factors, and microRNAs, which can regulate gene expression and modulate cellular processes involved in neural regeneration. These molecules can promote neuronal survival, axonal growth, and synapse formation, which are crucial for functional recovery. For example, exosome are transferred from Schwann cells to axons to benefit damaged nerves 88.

**Figure 3:**
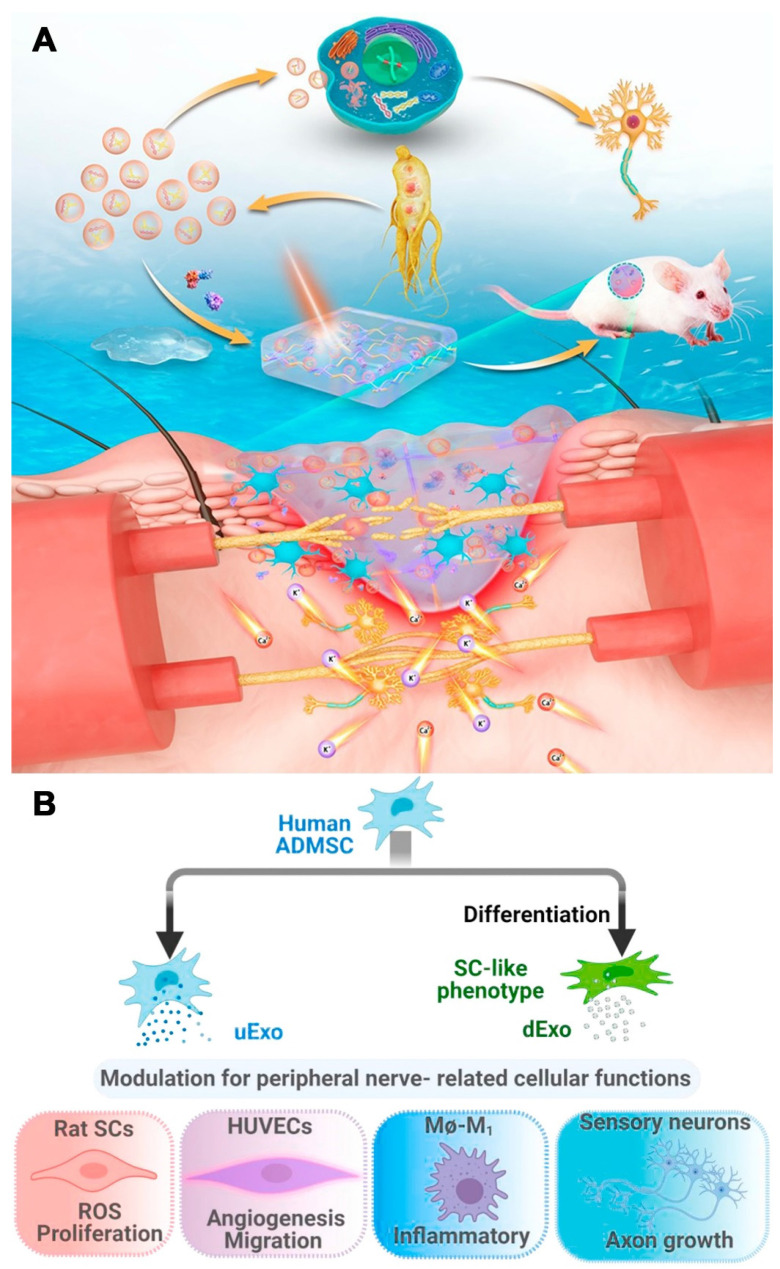
(A) Scheme of G-Exos stimulation in the neural differentiation of BMSCs resulting in sensory functions. Reproduced with permission from the ref. 90, Copyright 2021, ACS. (B) Exosomes (uExo and dExo) isolated from human ADMSCs and their SC-like (hADMSC-SCs) phenotypes are being studied for their ability to regulate peripheral nerve-related cellular processes. Reproduced with permission from the ref. 93, Copyright 2022, Elsevier (This work is licensed under CC BY-NC-ND 4.0. To view a copy of this license, visit http://creativecommons.org/licenses/by-nc-nd/4.0/).

Exosomes derived from medicinal plants have advantages over those derived from mammalian cells, including increased resources, reduced immunological risk/side effects, and cost-effective production. Xu et al. isolated G-Exos from ginseng juice. The authors tested the ability of these factors to transfer active nucleic acids to stem cells and influence the neural differentiation of BMSCs in vitro and in vivo. G-Exos are effective nanoplatform that deliver 20 G-E-miRs into BMSCs, proving that plant-derived Exos can transfer active plant nucleic acids to mammalian cells. Additionally, GSEA revealed that the G-Exos had increased levels of positive regulation of PI3K signaling genes, suggesting a potential mechanism and function of G-Exos. The transferred G-E-miRs could regulate 19 target genes related to neural differentiation, maturation, and functionalization in BMSCs (Figure 3 A) 90.

The integration of scaffolds and exosomes represents a promising, intricate approach to enhancing the resolution of traumatic brain injury. Liu et al. assessed the efficacy of a newly developed 3D-printed collagen/chitosan scaffold incorporated with exosomes derived from neural stem cells that were pretreated with insulin-like growth factor-1 (3D-CC-INExos) in enhancing the repair process and functional recovery following traumatic brain injury in rats. The Morris water maze test and modified neurological severity scores revealed that 3D-CC-INExos scaffold transplantation improved motor and cognitive functions in rats with traumatic brain injury. Immunofluorescence staining and transmission electron microscopy showed that 3D-CC-INExo implantation significantly improved nerve tissue recovery in the injured area 91.

Exosome and miRNA cargo modification is a promising treatment for peripheral nerve injury. Patients must sacrifice a normal nerve to collect patient-specific Schwann cell exosomes (SC Exos) to improve nerve regeneration. Evidence suggests that differentiated MSC-derived exosomes containing miRNAs can directly boost axonal regeneration or indirectly regulate inflammation to repair nerves. Exosomes derived from MSCs overexpressing miRNA cluster plasmids can improve nerve regeneration. To regenerate peripheral nerves, macrophage-derived exosomes containing miRNAs increased Schwann cell migration, proliferation, NGF, and laminin expression 92.

Liu et al. differentiated human adipose-derived MSCs (hADMSCs) into SC-like cells and compared their secreted growth factors to those of undifferentiated cells. Exosomes were then isolated from the supernatant of undifferentiated (uExos) and differentiated hADMSCs (dExos). HUVECs could internalize exosomes derived from peripheral nerve-related cells, such as SCs, endothelial cells, macrophages, and neurons. Exos increased rSC proliferation and ROS scavenging on Days 1 and 3. dExos increased HUVEC angiogenesis and decreased proinflammatory gene expression and cytokine secretion in M1 macrophages. Treatment with dExos significantly increased HiPSC-SN axonal growth. The miRNA profiles of hADMSC-derived exosomes and their parent cells were similar, and dExos contained more miRNAs than uExos. Upregulated miRNAs such as miRNA-132-3p and miRNA-199b-5p in dExos could promote neuroprotection, angiogenesis, and neuroimmune modulation (Figure 3 B) 93.

**Figure 4:**
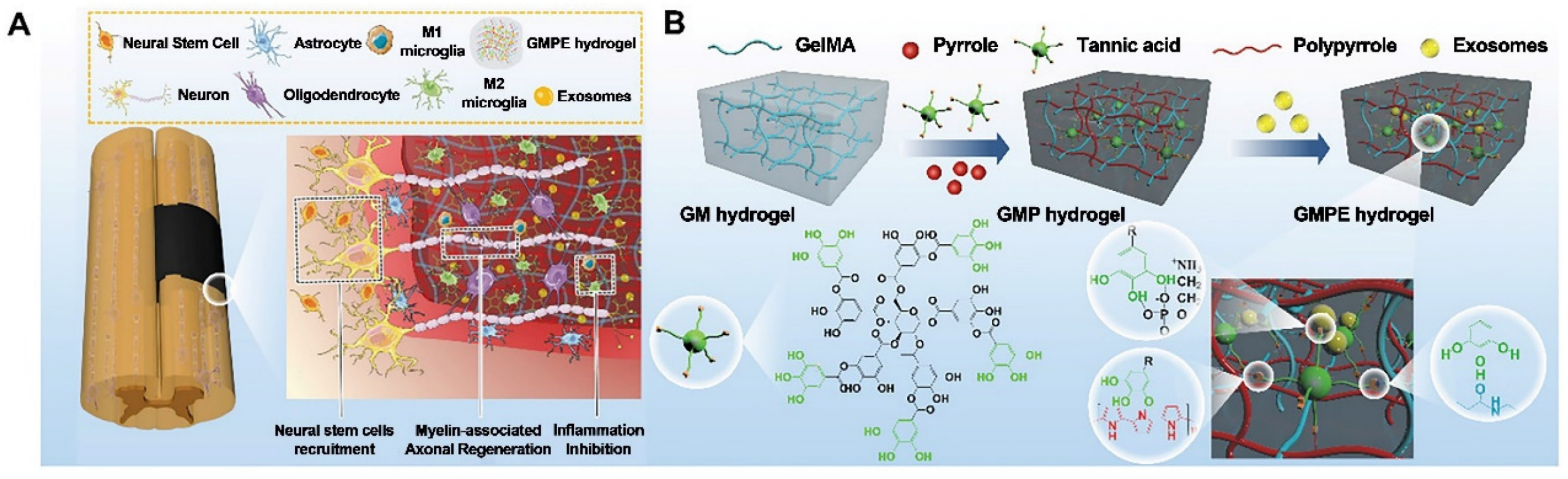
** (A)** GMPE hydrogel properties. The GMPE hydrogel reduces early inflammation, recruits NSCs, and promotes myelin-associated axonal regrowth to improve locomotor recovery following spinal cord hemisection. (B) GMPE hydrogel production was shown in three steps. TA reacting with the GM backbone amide bond and PPy chain nitrogen groups produced the GMP hydrogel. TA polyphenol groups and phosphate groups in exosome phospholipid formed hydrogen bonds to reversibly immobilize BMSC-exosomes inside GMP hydrogels. Reproduced with permission from the ref. 94, Copyright 2022, Wiley (This work is licensed under CC BY 4.0. To view a copy of this license, visit http://creativecommons.org/licenses/by/4.0/).

Due to their ability to promote regeneration and modulate immune reactions, BMSC-exosomes are a new cell-free therapeutic platform for treating various diseases. As promising nanocarriers, BMSC-exosomes carry therapeutic growth factors and miRNAs, enhancing axonal regeneration, angiogenesis, structural and electrophysiological improvements, and neuroinflammation, reducing gliosis and improving motor and sensory recovery after spinal cord injury (SCI). Fan et al. immobilized BMSC-exosomes in electroconductive hydrogels (GMPE) to inhibit inflammation, increase neural stem cell recruitment, and promote neuronal and myelin-associated axonal regeneration for SCI therapy. Due to the immunomodulatory properties of BMSC-exosomes, the GMPE hydrogel changed M1/M2 polarization from an M1-dominant to an M2-dominant phenotype via the NF-κB pathway. It also increased neuronal and oligodendrocyte differentiation in neural stem cells (NSCs) while inhibiting astrocyte differentiation and increasing axon outgrowth via the PTEN/PI3K/AKT/mTOR pathway (Figure 4 A-B) 94.

Zhang et al. used exosomes derived from mesenchymal stem cells obtained from the human umbilical cord (MExos) to deliver drugs. These MExos were incorporated into a scaffold that facilitated the migration of neural stem cells (NSCs) and served as a carrier for paclitaxel. A novel dual biospecific peptide (BSP) was used to retain MExos within the scaffold. The recruitment of endogenous neural stem cells (NSCs) by MExos to the site of injury, along with PTX-mediated induction of NSCs to generate neurons, enhanced motor functional recovery in rats following complete spinal cord injury (SCI). The use of this scaffold has been shown to improve neural regeneration while concurrently reducing the deposition of scar tissue 95.

While the potential of exosomes for neural regeneration is promising, additional research is needed to fully understand their mechanisms of action, optimize their therapeutic potential, and ensure their safety and efficacy. Clinical trials are underway to evaluate the use of exosomes in neurological disorders and injuries, and their use in regenerative medicine holds great potential for the future^34^.

### 4.2 Myocardial regeneration

Cardiac progenitor cells and mesenchymal stem cells have been extensively investigated as potential reservoirs of regenerative exosomes 96. In acute myocardial infarction, prolonged ischemia kills myocardial cells. This is the deadliest form of coronary artery disease. Myocardial infarction (MI) causes irreversible cardiomyocyte loss, left ventricular remodeling, and heart failure because adult heart cells cannot regenerate after ischemia. The prognosis of MI depends on irreversible cardiomyocyte and scar tissue loss 97. Immune pathway dysregulation, the suppression of postinfarction inflammation, spatial inhibition of the inflammatory response, and excessive fibrosis can also cause poor heart remodeling and heart failure.

**Figure 5:**
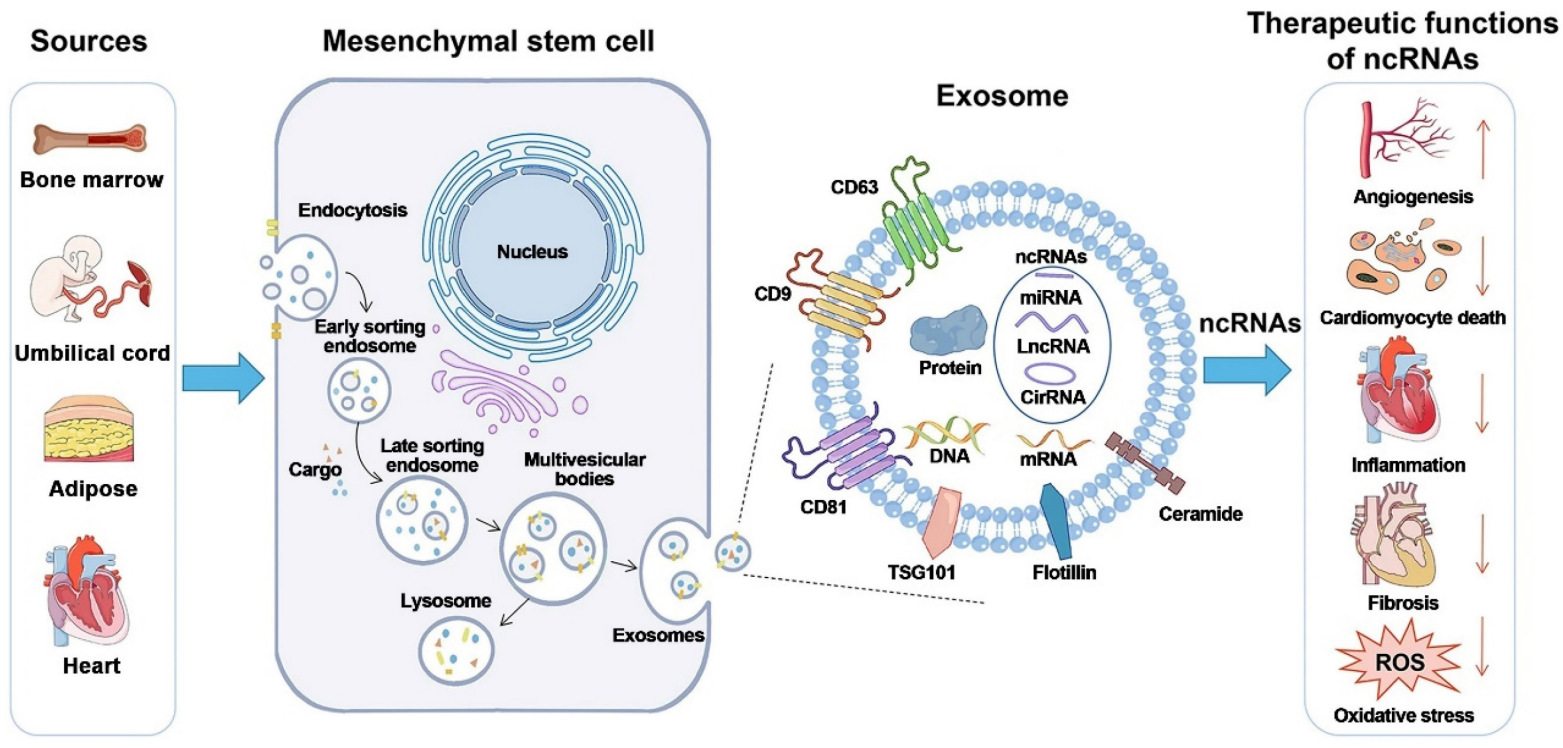
Exosome biogenesis and potential therapeutic activities of non-coding RNAs carried by mesenchymal stem cells. Exosome biogenesis is characterized by a twofold invagination of the plasma membrane and the production of intracellular multivesicular bodies (MVBs). For the first time, the plasma membrane invaginates and undergoes endocytosis, resulting in the formation of early sorting endosomes in the cytoplasm. Early sorting endosomes can develop into late sorting endosomes, which produce MVBs. MVBs are generated by the plasma membrane's second invagination, which can either merge with lysosomes or autophagosomes to be destroyed or fuse with the plasma membrane to release intraluminal vesicles (i.e., exosomes). CircRNA stands for circular RNA; LncRNA stands for long non-coding RNA; miRNA stands for microRNA; ncRNA stands for non-coding RNA; ROS stands for reactive oxygen species; and TSG101 stands for tumor susceptibility gene 101. Reproduced with permission from the ref. 99, Copyright 2021, Wiley (This work is licensed under CC BY 4.0. To view a copy of this license, visit http://creativecommons.org/licenses/by/4.0/).

Stem cell therapy has improved the treatment of MI, but transplanted cells rarely survive in the ischemic heart. Delivering the therapeutic components of these cells via exosomes can be effective 98. MSC and cardiac cell exosomes can be used to treat MI and other cardiovascular diseases in the future. The diversity of cardiac cells and their ability to mitigate or exacerbate myocardial injury necessitate further research on the effects of exosome cargo on specific signaling pathways (Figure 5). Future research should examine ways to pretreat or genetically modify parental cells to maximize exosome therapeutic efficacy 99.

Wang et al. prepared and thoroughly examined the functional role of ADSC-Exos containing miRNA-205 in MI injury and the molecular mechanism. They found that ADSC-Exos containing miRNA-205 could reduce myocardial fibrosis and inhibit apoptosis, which restored cardiac function in mice after MI injury. Additionally, ADSC-Exos containing miRNA-205 promoted angiogenesis. This study provides the primary evidence for the use of ADSC-Exos in the clinical treatment of MI 100.

**Figure 6:**
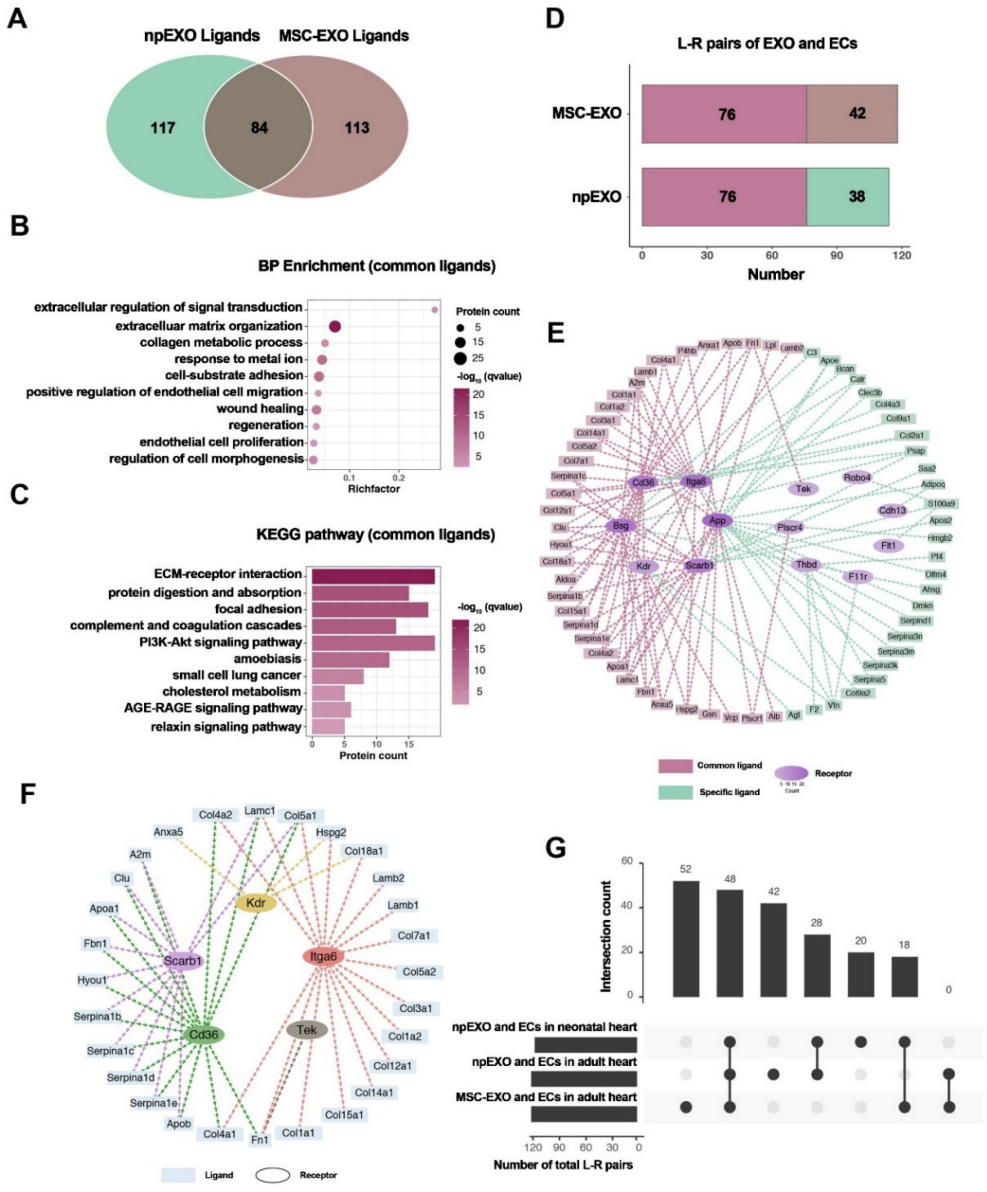
Identification of npEXO and MSC-EXO shared ligand-receptor pairings. (A) Venn diagram comparing total npEXO ligands to MSC-EXO ligands. (B) GO analysis (BP enrichment) of npEXO and MSC-EXO shared ligands. (C) KEGG pathway analysis of npEXO and MSC-EXO shared ligands. (D) The number of npEXO (or MSC-EXO) and ECs with L-R pairs in the adult heart (L-R pairs: ligand-receptor pairs; dark red denoted the standard part). (E) The communication network built between npEXO (or MSC-EXO) ligands and EC receptors in the adult heart (dark red represents the standard component and green represents the npEXO-specific part; the receptors' hierarchical color depth was related to the number of contacts). (F) The ligand-receptor interaction communication network overlapped between the common parts of the neonatal and adult heart, as well as another standard part shared by npEXO and MSC-EXO. (G) Venn diagram of ligand-receptor pairs in three groups (npEXO and ECs in the neonatal heart, npEXO and ECs in the adult heart, and MSC-EXO and ECs in the adult heart). Reproduced with permission from the ref. 101, Copyright 2023, MDPI (This work is licensed under CC BY 4.0. To view a copy of this license, visit http://creativecommons.org/licenses/by/4.0/).

Li et al. prepared npEXOs (neonatal mouse plasma exosomes) from neonatal mouse plasma that were 50-150 nm in diameter and had a bilayer-membrane structure. They delivered npEXOs to the adult heart after acute myocardial infarction (AMI) and found that these factors partially restored the heart. The results showed that npEXOs structurally and functionally facilitated cardiac repair after AMI by promoting CM survival and reducing cardiac remodeling. Cardiac endothelial cells (ECs) were identified as the primary noncardiomyocyte cell type that received ligand signals from nanoparticle-derived extracellular vesicles (npEXOs). This study revealed a ligand‒receptor network between npEXOs and cardiac endothelial cells (ECs). This network is believed to mediate the angiogenic effects induced by npEXOs and suggests potential targets for myocardial infarction (MI) therapy (Figure 6) 101.

Yuan et al. developed a gelatin-based biocompatible microneedle (MN) patch to deliver Exo/miR-29b mimic-containing exosomes derived from human umbilical cord MSCs (HUCMSCs) to prevent excessive cardiac fibrosis during MI treatment. This system integrated the benefits of the MN patch, exosomes, and miRNAs. After this patch was implanted in the infarcted region, the MN patch could retain more Exo/miR-29b mimic and release exosomes as the MNs dissolved. The exosomes could be internalized by cardiac fibroblasts to increase miR-29b expression, preventing myocardial fibrosis progression by inhibiting the TGF-β signaling pathway and preventing ECM remodeling. This composite patch reduced inflammation, infarct size, left ventricular wall thickness, and fibrosis in the risk region in mice, thereby facilitating cardiac repair after MI. This system combined the benefits of the MN patch, exosomes, and miRNAs and showed promise as an MI treatment 102.

Wang et al. used curcumin, bone marrow-derived mesenchymal stem cell-derived exosomes, and decellularized porcine cardiac extracellular matrix (dECM) hydrogels to create an injectable composite hydrogel with antifibrotic properties for MI treatment. The exosomes were preloaded with curcumin via electroporation. The hydrogels delivered curcumin-encapsulated exosomes to reduce cardiac fibrosis after MI. Exosomes and dECM hydrogels have unique bioactivity for cardiac repair and enhance curcumin solubility and sustained release in the infarcted myocardium. In vitro experiments showed that Exos/Cur were internalized by fibroblasts and prevented myofibroblast transformation, thereby preventing fibrosis. Injection of the composite hydrogels into mice with MI increased angiogenesis, decreased the infarct size, thickened the left ventricular wall, and inhibited fibrosis, thereby facilitating cardiac repair. This study combined the benefits of natural biomaterials (dECM), extracellular vesicles (exosomes), and an antifibrotic molecule (curcumin) to develop a clinically promising therapeutic system to treat myocardial fibrosis after MI 103.

**Figure 7:**
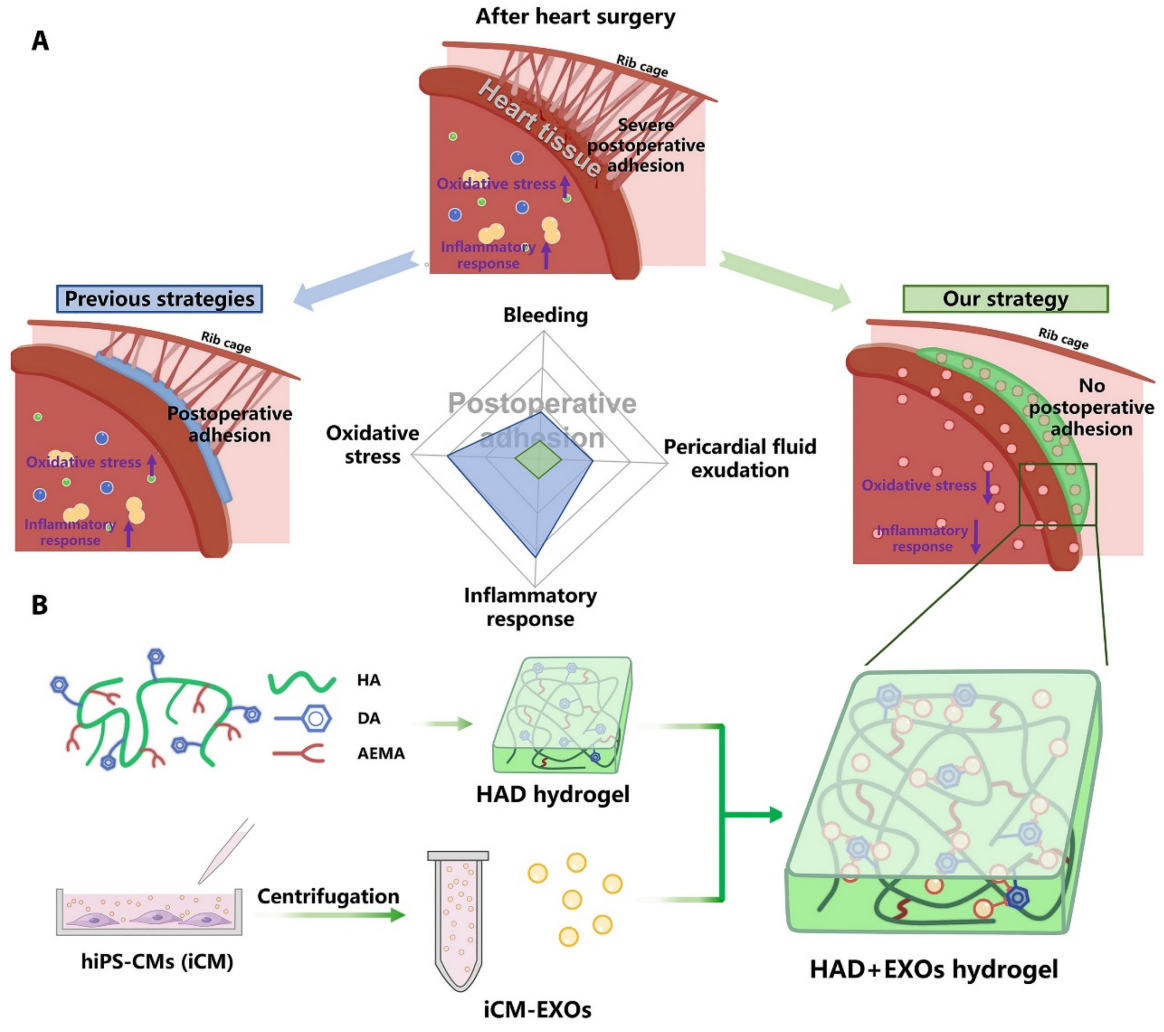
HAD+EXOs hydrogel overview for preventing postoperative adhesion following cardiac surgery. (A) Illustration of postoperative pericardial adhesion. Severe postoperative pericardial adhesion is related to bleeding, pericardial fluid exudation, oxidative stress, and an inflammatory response. Previously, hydrogels or film barriers were used to stop bleeding or pericardial fluid exudation. Nonetheless, these treatments did not diminish oxidative stress or the inflammatory response. (B) We took use of the catechol groups in DA to develop an injectable, photocurable Janus HAD hydrogel with asymmetric adhesion and the ability to produce sustained release of hiPSC cardiomyocyte-derived exosomes that reduce oxidative stress and inflammation. In this way, the HAD and exosomes work together to avoid or reduce postoperative pericardial adhesion. Reproduced with permission from the ref. 104, Copyright 2023, AAAS (This work is licensed under CC BY-NC 4.0. To view a copy of this license, visit http://creativecommons.org/licenses/by-nc/4.0/).

Wang et al. encapsulated induced pluripotent stem cell-derived cardiomyocyte-derived exosomes (iCM-EXOs) in asymmetric HAD (hyaluronic acid-*g*-(2-aminoethyl methacrylate hydrochloride-dopamine)) hydrogels to create HAD+EXO hydrogels that could reduce oxidative stress and pericardial adhesion after cardiac surgery. The UV-photo-crosslinked HAD hydrogel had one side that facilitated adhesion to the wet myocardial surface and the other that resisted adhesion to the thoracic cavity. The adhesion-resistant side of the HAD+EXO hydrogel could prevent the recruitment of GATA6+ cavity macrophages from the thoracic cavity, thereby protecting the iCM-EXOs from macrophages. Because of the high affinity of the catechol group for exosomes, the adhesion side of the HAD+EXO hydrogel could locally sustain the release of iCM-EXOs to alleviate oxidative stress. By inhibiting Nrf2 activation, iCM-EXOs significantly reduced oxidative stress in H_2_O_2_-treated primary cardiomyocytes in vitro. After 15 days, the HAD+EXO hydrogel exhibited sustained release of the encapsulated iCM-EXOs, and the substantial release of the iCM-EXOs from the HAD+EXOs alleviated oxidative stress and reduced inflammation in a rat model after cardiac surgery. In vivo, the HAD+EXO hydrogel significantly reduced pericardial adhesion but had no adverse effects on cardiac function. The HAD component helped to form a physical barrier, while iCM-EXOs exhibited antioxidant properties. The authors showed that the HAD+EXO hydrogel was a promising novel agent for reducing postoperative pericardial adhesion (Figure 7) 104.

In summary, exosomes derived from regenerative cell types such as cardiac progenitors and mesenchymal stem cells represent a promising new approach to stimulating myocardial regeneration after injury. However, additional research is needed to determine the full potential of these materials for cardiac repair 105,106. Challenges remain in exosome isolation methods, the optimization of exosome cargo, scaling production, and improving delivery/retention. However, preclinical studies have demonstrated the feasibility of using exosomes as therapeutic agents for myocardial regeneration.

### 4.3 Hepatic regeneration

Liver disease has emerged as a significant global health and economic challenge due to its extensive range of ailments, diverse etiologies, and intricate therapeutic interventions. Most hepatic disorders progress toward the terminal stage of hepatic dysfunction, which is characterized by the substantial accumulation of extracellular matrix. This excessive matrix deposition poses a formidable obstacle for the use of effective regenerative processes to treat the liver and constituent hepatocytes 107.

Liver transplantation represents the only therapeutic option for individuals afflicted with end-stage liver disease. However, the scarcity of appropriate organ donors, exorbitant treatment expenses, and surgical complications significantly diminish overall survival rates. Hence, it is imperative to develop a highly effective treatment modality. The investigation of cell-free therapy has emerged as a prominent area of focus within regenerative medicine. Exosomes derived from mesenchymal stem cells (MSCs) possess regulatory properties and can transport functional molecules across physiological barriers to effectively communicate with and regulate target cells. These exosomes exhibit a minimal propensity for tumorigenesis. Exosomes derived from mesenchymal stem cells (MSCs) can stimulate the proliferation of hepatocytes and facilitate the restoration of impaired liver tissue 108. This is achieved through intercellular communication and the modulation of signal transduction pathways. Consequently, the potential of MSC-derived exosomes has led to the development of novel therapeutic approaches for liver diseases 109.

Studies have shown that exosomes can promote hepatic regeneration through several mechanisms. Exosomes have also been studied for their potential role in hepatic regeneration, including liver tissue repair and regrowth after injury or damage. The liver has a remarkable ability to regenerate, and exosomes have been shown to contribute to this process; they can stimulate the proliferation of hepatocytes, which are the primary functional cells of the liver and are crucial for tissue repair 110,111. Exosomes can also enhance the migration of liver progenitor cells, which are involved in liver regeneration and differentiation. Exosomes derived from various cell types, including stem and liver cells, have been investigated for their regenerative properties in liver diseases and injuries. These exosomes can be isolated from the culture media of these cells and then administered to the liver. Furthermore, exosomes can transfer various bioactive molecules, such as growth factors, cytokines, and microRNAs, to recipient cells in the liver 112. These molecules can regulate gene expression, promote cell survival, and modulate the immune response, which is essential for hepatic regeneration 113.

**Figure 8:**
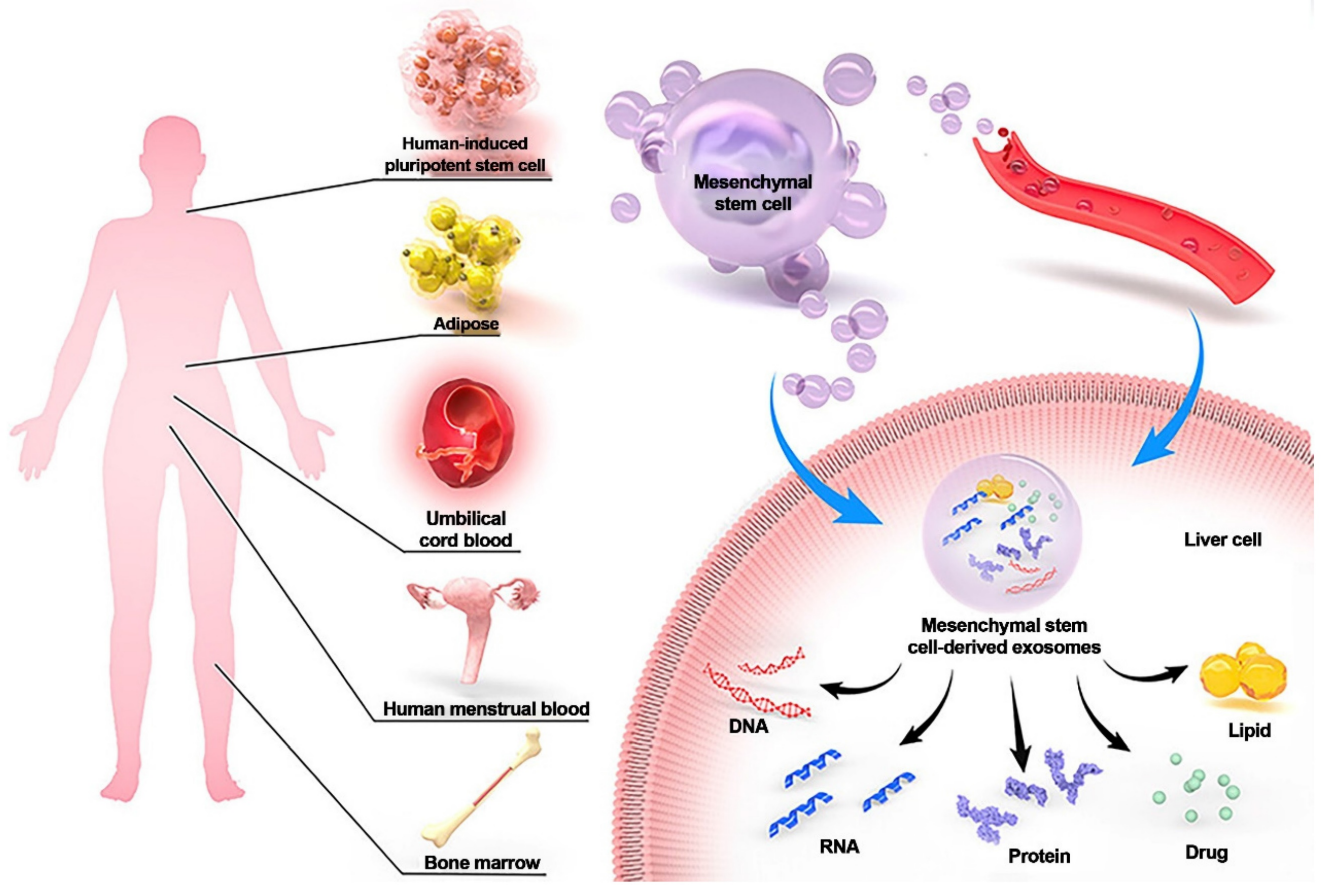
MSC-Exos have demonstrated delivery vehicle potential and the potential to treat liver disorders. MSCs can be extracted from a variety of adult tissues, including bone marrow, umbilical cord blood, adipose tissue, and human menstrual blood. MSC-Exos helps the liver by secreting DNA, RNA, protein, lipids, and drugs via paracrine and blood transport. Reproduced with permission from the ref. 114, Copyright 2023, Dove Medical Press (This work is licensed under CC BY-NC 4.0. To view a copy of this license, visit http://creativecommons.org/licenses/by-nc/4.0/).

Exosomes derived from specific cell types, such as mesenchymal stem cells or liver progenitor cells, have shown promising results in preclinical studies and early clinical trials for liver diseases such as liver fibrosis, cirrhosis, and acute liver injury. For example, mesenchymal stem cells (MSCs) can be used as an alternative therapy for liver disease, especially cirrhosis, liver failure, and complications from liver transplantation. On the other hand, MSCs have the potential to be tumorigenic. Exosomes derived from MSCs (MSC-Exos), which can mediate intercellular communication for MSCs, contain various proteins, nucleic acids, and DNA. MSC-Exos can treat liver diseases in multiple ways, including regulating immunity, inhibiting apoptosis, promoting regeneration, and drug delivery. MSC-Exos can prevent and treat liver injury, fibrosis, cancer, and other diseases via signal transduction, immune regulation, tissue regeneration promotion, drug delivery, and other mechanisms. However, the mechanism by which MSC-Exos treat liver disease is not fully understood, since preclinical studies are insufficient, and the clinical application of these materials faces several challenges (Figure 8) 114.

**Figure 9:**
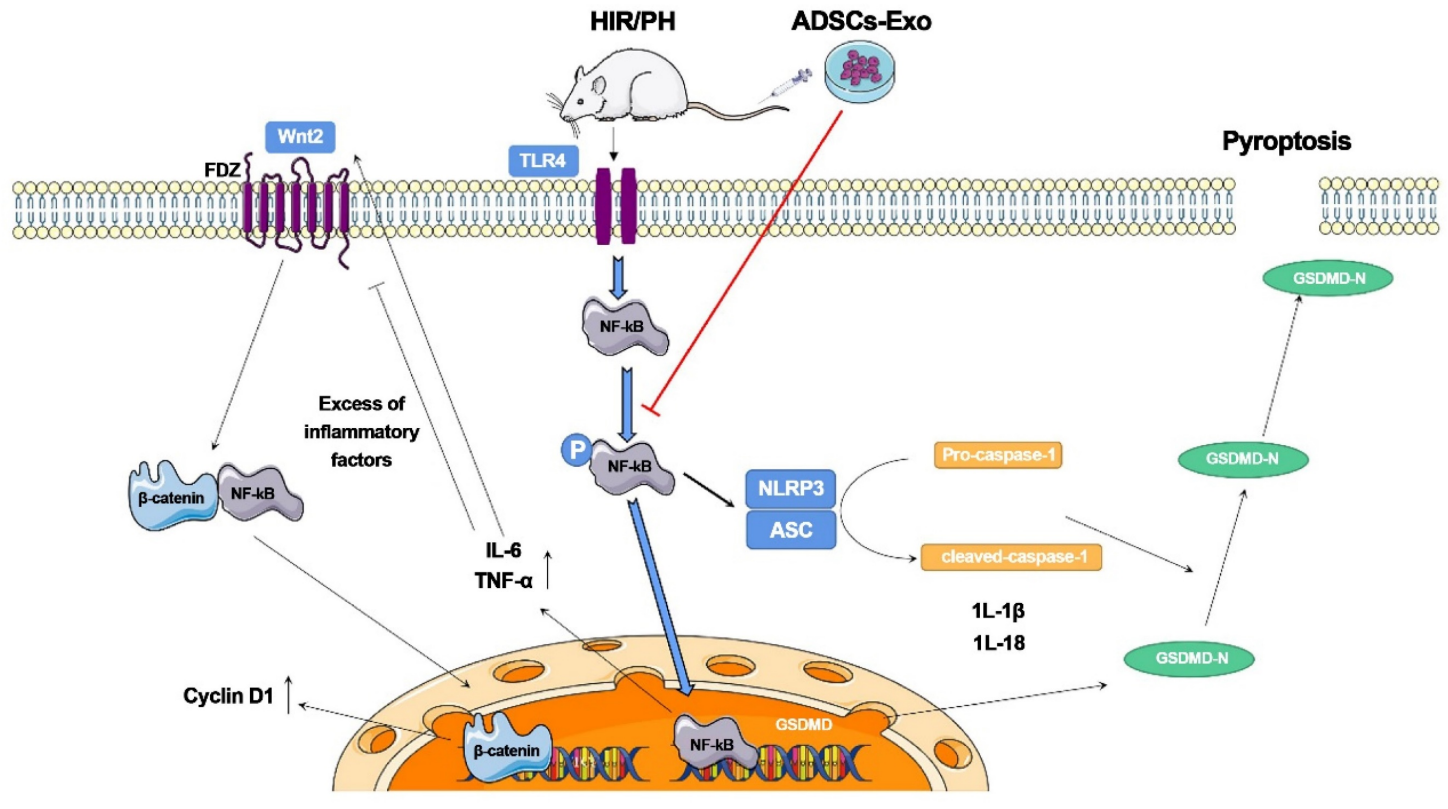
The ADSCs-Exo pathway reduces pyroptosis and improves tissue regeneration in the liver, which has been injured by ischemia-reperfusion and partial resection. The findings reveal that ADSCs-Exo suppresses the NF-κB signaling pathway while activating the Wnt/β-catenin signaling pathway. If the liver is injured severely, an inflammatory reaction occurs, which inhibits hepatocyte proliferation. After transplanting ADSCs-Exo, NF-κB phosphorylation is blocked, and the creation of inflammation and activation of GSDMD is not stimulated. ADSCs-Exo, on the other hand, can activate the Wnt/β-catenin pathway, and -catenin translocates into the nucleus, enhancing Cyclin D1 expression and driving cell proliferation. Finally, ADSCs-Exo helps to prevent cell injury while also promoting tissue regeneration and functional recovery. Reproduced with permission from the ref. 115, Copyright 2022, MDPI (This work is licensed under CC BY 4.0. To view a copy of this license, visit http://creativecommons.org/licenses/by/4.0/).

Piao et al. 115 investigated the beneficial effects of exosomes derived from adipose-derived mesenchymal stem cells (ADSC-Exos) on rats after hepatic ischemia‒reperfusion with partial resection injury. They discovered that postoperative tail vein injection of ADSC-Exos could effectively inhibit the expression of pyroptosis-related factors, such as NLRP3, ASC, caspase-1, and GSDMD-N, and promote the expression of regeneration-related factors, such as Cyclin D1 and VEGF. Furthermore, these cellular activities were linked to the NF-κB and Wnt/β-catenin signaling pathways. According to these findings, ADSCs and ADSC-Exos could reduce pyroptosis in the injured liver and promote the expression of liver regeneration factors, thereby inhibiting the NF-κB pathway and activating the Wnt/β-catenin pathway. Although adipose-derived mesenchymal stem cell (ADSC) transplantation can reduce liver injury, it causes a significant increase in the expression of the pyroptosis-related protein GSDMD-N. This study indicates that ADSC-Exos have distinct advantages as a cell-free therapy to replace stem cells and have broad research potential in the clinical diagnosis and treatment of liver injuries (Figure 9).

**Figure 10:**
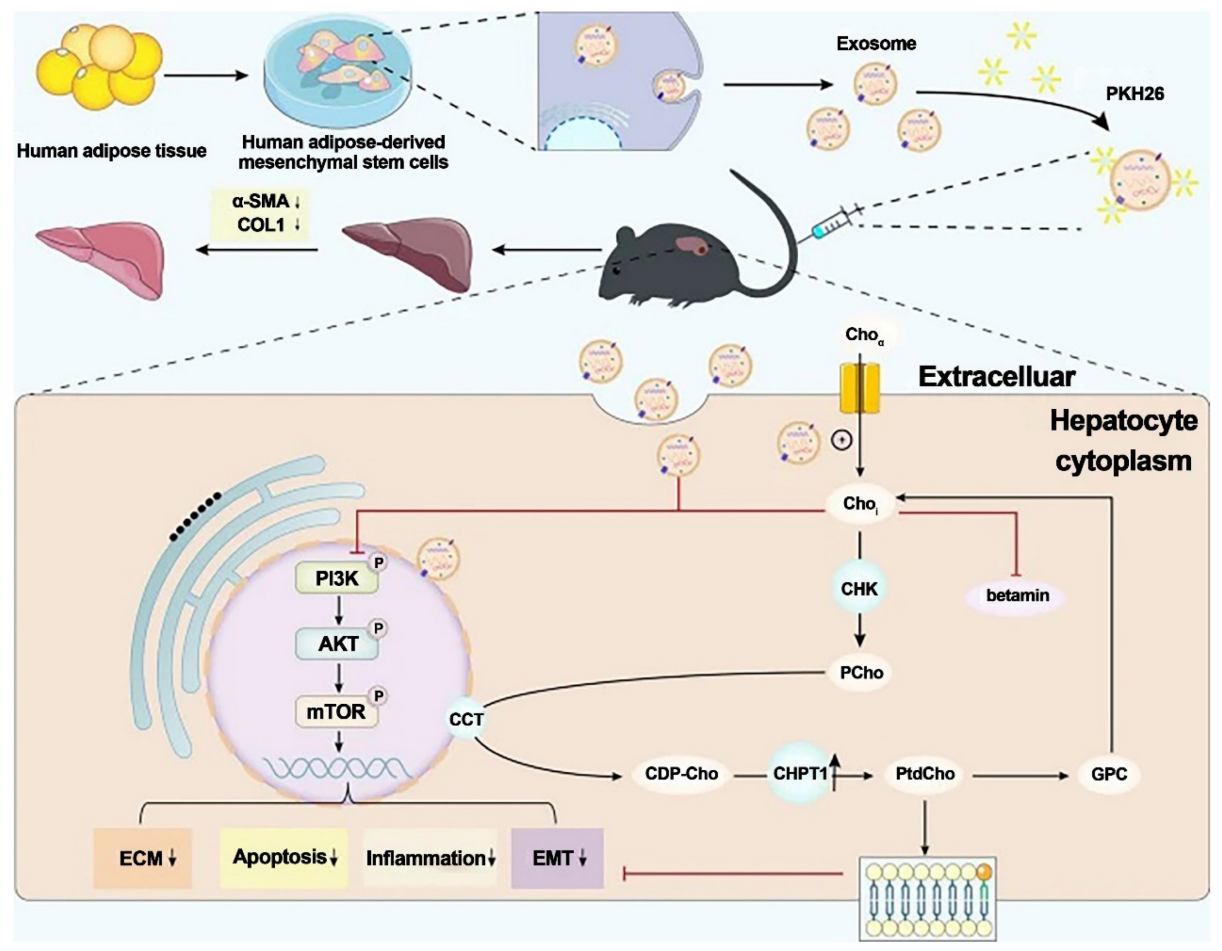
Exosomes generated from human adipose mesenchymal stem cells reduce hepatic fibrosis by suppressing the PI3K/Akt/mTOR signaling pathway and altering choline metabolism. Reproduced with permission from the ref. 116, Copyright 2023, Springer Nature (This work is licensed under CC BY 4.0. To view a copy of this license, visit http://creativecommons.org/licenses/by/4.0/).

Liver fibrosis is a chronic liver disease characterized by a progressive wound-healing response induced by liver injury. There are currently no approved therapies for liver fibrosis. Exosomes derived from human adipose mesenchymal stem cells (hADMSCs-Exos) have shown promising therapeutic effects in treating liver diseases (Figure 10) 116.

Zhang et al. investigated the antifibrotic effects of hADMSC-Exos (exosomes isolated from hADMSCs) in vitro and in vivo. They discovered that hADMSC-Exos could effectively inhibit the expression of profibrogenic proteins and epithelial-to-mesenchymal transition (EMT) in activated hepatic stellate cells by exacerbating apoptosis and arresting cells in the G1 phase in a dose-dependent manner. Omics analysis revealed that the mechanism by which hADMSC-Exos ameliorated hepatic fibrosis involved inhibiting the PI3K/AKT/mTOR signaling pathway, which affected metabolite changes in lipid metabolism and primarily regulated choline metabolism. The activation of CHPT1 by hADMSC-Exos aided in the formation and maintenance of vesicular membranes. These findings shed light on the molecular mechanisms underlying the antifibrotic effects of hADMSC-Exos, emphasized metabolic homeostasis and will aid in the development of safe and effective therapeutics. As a result, this study is an essential step toward the clinical use of hADMSC-Exos to treat chronic liver fibrosis.

It is important to note that the field of exosome-based therapies for hepatic regeneration is still in its early stages, and additional research is needed to fully understand the underlying mechanisms, optimize the therapeutic potential of these agents, and ensure their safety and efficacy.

### 4.4 Wound regeneration

The skin is the largest organ in humans and serves as a physical barrier, mediates immune defense and thermoregulation, and acts as a tactile sensor 117,118. Wound healing is a complex process that involves inflammation, cell migration, proliferation, and tissue remodeling 57,61,118. Exosomes have been shown to contribute to wound healing by promoting cell migration, reducing inflammation, and stimulating tissue regeneration 57,119-122. The paracrine effects of MSC-exosomes, which are the primary bioactive extracellular vesicles produced by MSCs, has been proposed as a new potential cell-free approach for wound healing and skin regeneration 123. MSC-derived exosomes (MSC-Exos) are promising nanomaterials for wound healing. MSC-Exos protect skin cells from severe damage and restore their function under adverse conditions 124.

Exosomes promote wound healing through several mechanisms. They can stimulate the migration of various cell types involved in wound healing, such as fibroblasts and endothelial cells. Exosomes also contain growth factors and cytokines that promote cell proliferation and tissue regeneration 125. Additionally, they can modulate the immune response, reduce inflammation and promote a favorable environment for wound healing 35. For example, Exos can ameliorate type 2 diabetes by reversing peripheral insulin resistance and mitigating β-cell destruction. This leads to the restoration of insulin secretion and a reduction in blood glucose levels. Thus, Exo therapy for diabetic wounds has distinct advantages because of its ability to facilitate wound closure and ameliorate the diabetic conditions of individuals, thereby establishing a more conducive milieu for wound healing 126.

**Figure 11:**
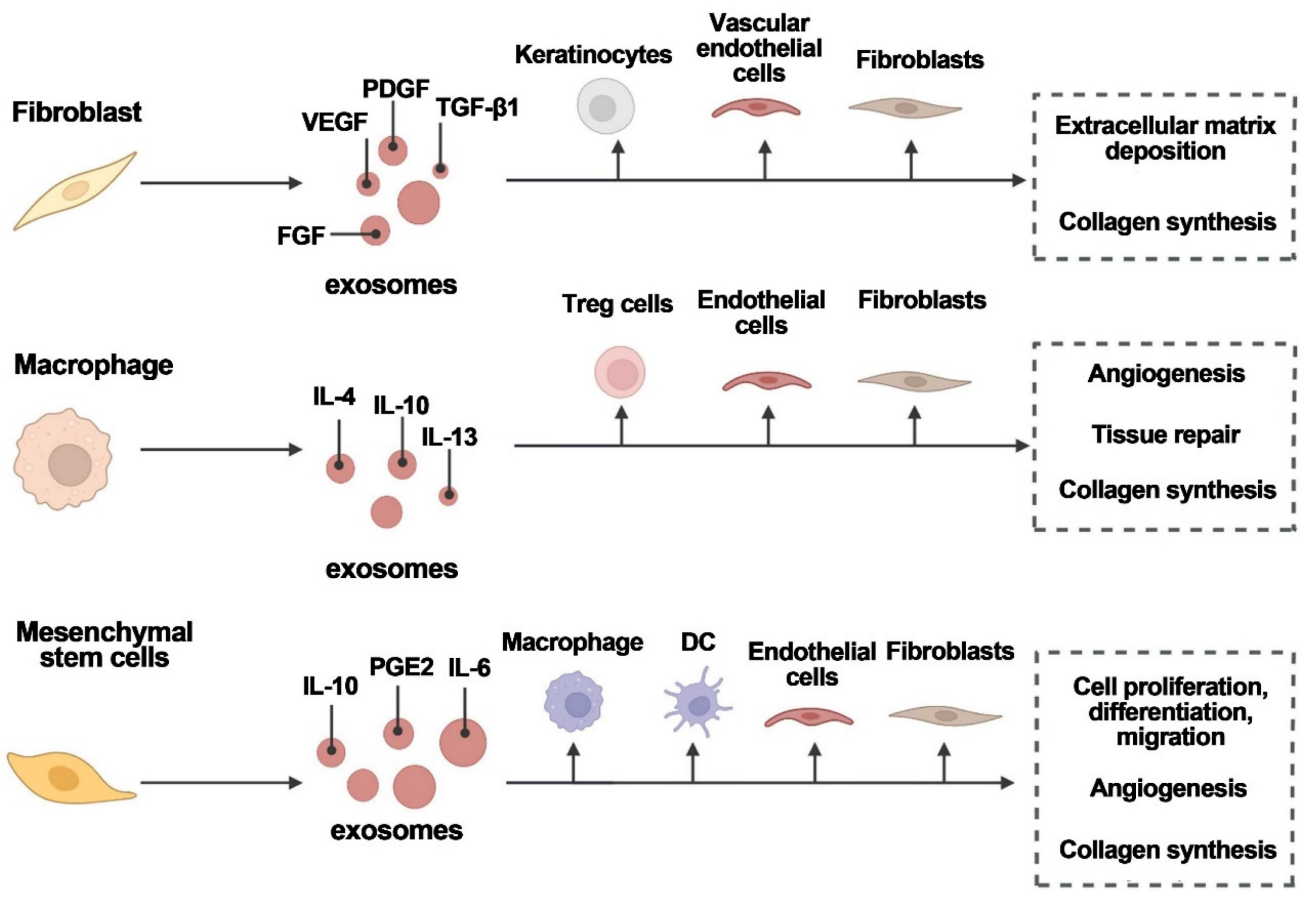
Various cell types secrete exosomes to aid in wound healing. Exosomes are released by fibroblasts, macrophages, and mesenchymal stem cells, which act on target cells to promote angiogenesis, tissue repair, and wound healing. FGF is fibroblast growth factor; IL is interleukin; PDGF is platelet-derived growth factor; PGE2 is prostaglandin E2; TGF-1 is transforming growth factor-1; and VEGF is vascular endothelial growth factor. Reproduced with permission from the ref. 16, Copyright 2023, Elsevier (This work is licensed under CC BY-NC-ND 4.0. To view a copy of this license, visit http://creativecommons.org/licenses/by-nc-nd/4.0/).

Furthermore, exosomes can transfer genetic material, such as microRNAs, to recipient cells, regulating gene expression and promoting wound healing processes (Figure 11) 16. However, poor targeting and easy removal of exosomes from wounds are significant barriers to their clinical application. Thus, bioengineering technology has been used to modify exosomes, allowing for higher concentrations of exosomes and the construction of more stable particles with specific therapeutic capabilities. Using biomaterials loaded with exosomes, such as hydrogels, patches and microneedles, could be a promising strategy to increase doses, achieving therapeutic efficacy, and maintaining sustained release 127-129.

Lee et al. investigated the effects of ASC-derived exosomes (ASC-EXOs) on human dermal fibroblasts (HDFs) and how they might be combined with hyaluronic acid (HA) in wound healing and dermal filler models in vivo. They found that ASC-EXOs increased proliferation and migration in HDFs; in addition, ASC-EXOs stimulated collagen production in HDFs by upregulating the expression of genes involved in cell proliferation and wound healing. Furthermore, the combination of HA and ASC-EXOs improved wound healing and tissue remodeling, and ASC-EXO treatment increased proliferation, wound healing gene expression, and collagen production in HDFs, suggesting the possibility of ECM remodeling. Overall, the results of this study indicate that treatment with ASC-EXOs, alone or in combination with HA, can be used as a novel therapeutic approach for wound healing and tissue regeneration in various clinical settings 130.

**Figure 12:**
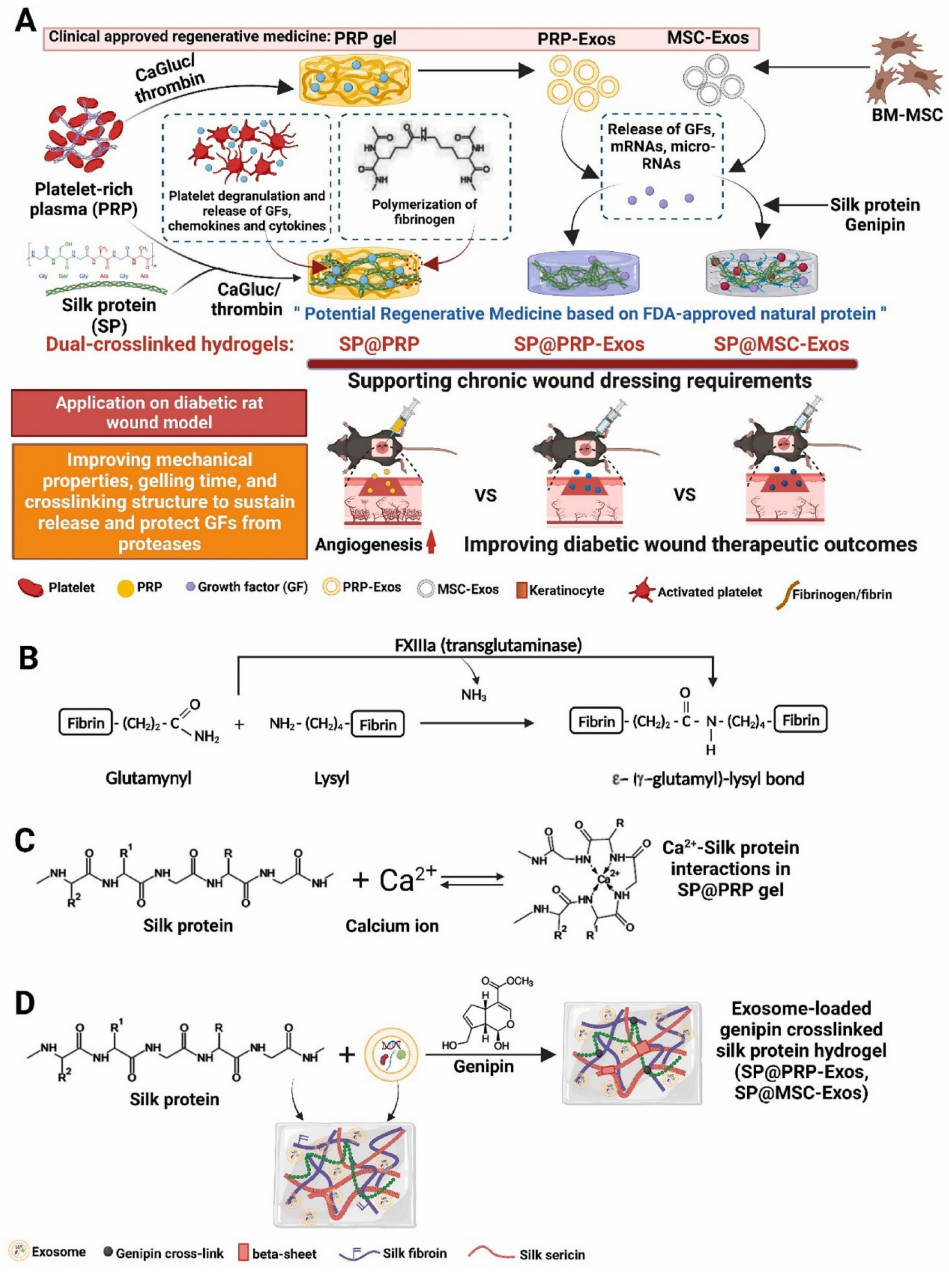
(A) Schematic representation of the synthetic process for dual-crosslinked hydrogels (SP@PRP, SP@PRP, and SP@PRP) and their application as diabetic wound dressings; (B) Factor XIIIa catalyzes a chemical reaction that results in highly covalently crosslinked insoluble fibrin via glutamine and lysine residues; (C) Ca^2+^-silk protein interactions in SP@PRP activated with calcium gluconate; and (D) formation of exosome-loaded genipin-crosslinked silk protein hydrogels. Reproduced with permission from the ref. 131, Copyright 2023, Elsevier (This work is licensed under CC BY-NC-ND 4.0. To view a copy of this license, visit http://creativecommons.org/licenses/by-nc-nd/4.0/).

Platelet-rich plasma (PRP) gels, PRP-derived exosomes (PRP-Exos), and mesenchymal stem cell-derived exosomes (MSC-Exos) have all shown promise in the treatment of wounds 132. Bakadia et al. 131 created novel dual-crosslinked silk protein (SP) (sericin and fibroin) hydrogels, including SP@PRP, SP@MSC-Exos, and SP@PRP-Exos, to synergistically promote diabetic wound healing. SP@PRP was created by combining PRP and SP with calcium gluconate/thrombin as an agonist, whereas SP@PRP-Exos and SP@MSC-Exos were created by combining exosomes and SP with genipin as a crosslinker. SP improved the mechanical properties of the wound and allowed for the sustained release of growth factors (GFs) and exosomes, which overcame the limitations of PRP and exosomes in wound healing (Figure 12). The dual-crosslinked hydrogels exhibited shear-induced thinning, self-healing, and microbial biofilm eradication. Furthermore, the dual-crosslinked hydrogels inhibited the growth of *E. coli*, *P. aeruginosa*, *S. aureus*, and MRSA. In a diabetic wound model, adding SP to PRP, PRP-Exos, or MSC-Exos improved the overall mechanical properties of the hydrogels. It prolongs the release of bioactive substances such as VEGF and TGF-1, resulting in a cascade of beneficial biological events and increasing tissue regeneration. By upregulating GF expression and downregulating matrix metalloproteinase-9 expression, the dual-crosslinked hydrogels inhibited NETosis and oxidative stress, induced M2 polarization, granulation tissue matrix formation, neovascularization, sebaceous gland and hair follicle regeneration, and increased collagen deposition, all of which are beneficial for re-epithelialization and scar-free wound closure. These dual-crosslinked hydrogels could be promising bioactive wound dressings for treating and managing chronic wounds, including diabetic wounds infected with bacteria.

Huang et al. proposed a novel herringbone microfluidic bioreactor with cell microcarrier integration for the batch preparation of stem cell exosomes. In this system, bone mesenchymal stem cells (BMSCs) and NIH-3T3 fibroblasts (FBs) were chosen as working cells. The microfluidic device had parallel herringbone microgrooves on top and a microcolumn array on the bottom, which provided a controllable fluidic environment for stem cells cultured on gelatin methacryloyl (GelMA) microcarriers and exosome secretion by these cells; moreover, the yield of exosomes produced by this bioreactor was significantly higher than that produced by traditional flask cell culture. By dynamically adjusting the perfusion rate of the medium, the microfluidic bioreactor produced approximately 21-fold more exosomes than static flask culture. Moreover, the bioreactor was accessible to various cells and could be used to harvest many exosomes. Furthermore, the function of exosomes in wound healing in vivo, including the use of exosomes in combination with other treatments, was verified by examining the intrinsic benefits of exosomes, such as immune regulation, activity maintenance, and biocompatibility. Microfluidic and microcarrier-based platforms are efficient for harvesting exosomes and have excellent potential for wound healing and other biomedical applications 133.

It has been reported that pretreating MSCs with chemical or biological factors improves the biological activities of MSC-derived exosomes. Li et al. investigated whether exosomes derived from human umbilical cord MSCs (hucMSCs) that were preconditioned with Nocardia rubra cell wall skeleton (Nr-CWS) had superior proangiogenic effects on diabetic wound repair and the molecular mechanisms. The findings revealed that Nr-CWS-Exos aided endothelial cell proliferation, migration, and tube formation in vitro. Compared to Exos, Nr-CWS-Exos significantly promoted wound healing by facilitating wound tissue angiogenesis. Furthermore, the role of circRNAs in wound repair mediated by Nr-CWSExos was investigated, and the results revealed that circIARS1 expression was increased after Nr-CWS-Exo treatment of HUVECs. CircIARS1 increased the proangiogenic effects of Nr-CWS-Exos on endothelial cells through the miR-4782-5p/VEGFA axis. Exosomes derived from Nr-CWS-pretreated MSCs could serve as agents for diabetic wound treatment by advancing the biological function of endothelial cells via the circIARS1/miR-4782-5p/VEGFA axis. The researchers found that overexpression of miR-4782-5p inhibited circIARS1-mediated angiogenesis. These findings showed that Nr-CWS preconditioning facilitated the ability of MSC-Exos to enhance angiogenesis by upregulating the circIARS1/miR-4782-5p/VEGFA axis, thereby accelerating diabetic wound healing. These findings offer a promising cell-free therapeutic strategy for diabetic wound treatment 134.

Evidence suggests that the delivery of circRNAs via adipose-derived stem cell (ADSC)-derived exosomes (ADSC-Exos) may be a strategy for diabetic wound repair. However, the drawbacks of exosomes, such as the rapid loss of biological activity and unknown biological mechanisms, limit their clinical application. Hu et al. created hypoxia-pretreated ADSC-Exo (ADSC-HExo)-embedded GelMA hydrogels (GelMA-HExo) through noncovalent interactions and physical embedding. In response to illumination, these materials quickly converted into a gel state, adapting to the irregular diabetic wounds. The GelMA-HExo hydrogels had a loose porous structure in vitro and stable degradation and expansion rates. GelMA-HExo hydrogels promoted wound healing in diabetic mice in vivo. In particular, ADSC-HExos had a positive therapeutic effect by increasing circ-Snhg11 expression. This study revealed that ADSC-HExos could deliver circ-Snhg11, which improved survival and EC function, possibly by activating miR-144-3p/NFE2L2/HIF1 signaling. In conclusion, this study examined the effects of hypoxic engineered exosome hydrogels and downstream targets on diabetic wound repair. These hydrogels are expected to be a novel clinical treatment approach with potential applications in other disease areas 135.

Overall, exosomes have shown promising potential in wound healing and tissue regeneration. However, further research is still needed to fully understand the underlying mechanisms and optimize the therapeutic applications of these substances.

### 4.5 Bone regeneration

Exosomes have also been widely investigated for their potential role in bone regeneration, which refers to restoring bone tissue after injury, disease, or skeletal defects 136-138. Bone regeneration is a complex process involving the recruitment and differentiation of various cell types and the deposition of new bone matrix 139,140. Various cell types, including osteoblasts, osteoclasts, osteocytes, and stem cells, govern the regulation of bone metabolism. Effective treatments that promote bone formation and regeneration are necessary to manage pathologies such as osteoporosis, osteoarthritis, osteonecrosis, and traumatic fractures 141-145.

Exosomes derived from different cell types, such as mesenchymal stem cells (MSCs) or osteoblasts, have been studied for their regenerative effects on bone tissue 147,148. These exosomes can be isolated from the culture media of these cells and subsequently applied to the site of the bone injury or defect. Studies have shown that exosomes can promote bone regeneration through several mechanisms. They can stimulate the proliferation and differentiation of osteoblasts, which are responsible for bone formation. Exosomes can also enhance the recruitment and differentiation of mesenchymal stem cells into osteoblasts, further contributing to bone regeneration.

**Figure 13:**
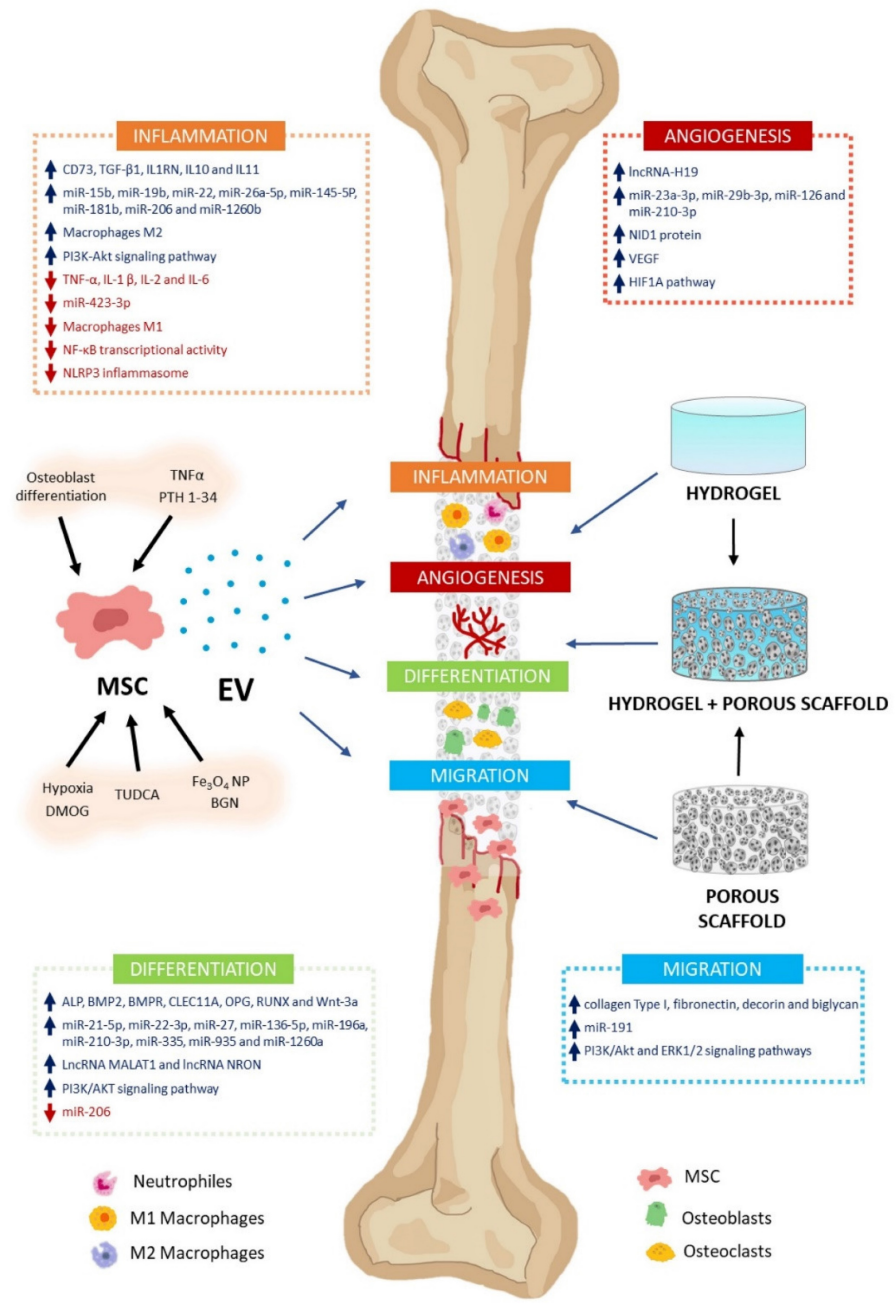
The MSC-EV effect on bone regeneration. MSC-EV can regulate bone formation and regeneration. These include inflammation, angiogenesis, differentiation, and cell migration. Their cargos contain varying levels of biologically active molecules (e.g., growth factors, cytokines, miRNA, lncRNA). They activate or inhibit several signaling pathways involved in these processes. MSC cultures can be preconditioned with various molecules (TNFα, PTH 1-34, TUDCA, DMOG), nanoparticles (Fe3O4 NP, BGN), or culture conditions (osteoblast differentiation, hypoxia) to encourage high-regenerative EV secretion. Using biomaterials as vehicles and delivery systems, MSC-EV can treat bone defects. Scaffolds facilitate cell migration, proliferation, and differentiation, enabling bone regeneration. Hydrogels, porous scaffolds, and hybrids are promising biomaterials for bone damage treatment. Reproduced with permission from the ref. 146, Copyright 2023, MDPI (This work is licensed under CC BY 4.0. To view a copy of this license, visit http://creativecommons.org/licenses/by/4.0/).

Recent approaches in bone regeneration include cell therapy involving mesenchymal stem cells (MSCs). Mesenchymal stem cells (MSCs) can differentiate into osteoblasts, which are responsible for bone formation 149. However, the regenerative capabilities of MSCs are at least partly affected by their paracrine properties. The primary mechanisms involved in these processes include extracellular vesicle (EV) secretion. Extracellular vesicles (EVs) significantly regulate various regenerative processes, including inflammation, angiogenesis, cell proliferation, migration, and differentiation (Figure 13) 146.

Exosomes contain various bioactive molecules, including growth factors, cytokines, and microRNAs, which can regulate gene expression and modulate cellular processes involved in bone regeneration 150. These molecules can promote angiogenesis (the formation of new blood vessels), reduce inflammation, and enhance the deposition of new bone matrix. Furthermore, exosomes can also carry genetic material, transferring specific microRNAs or other regulatory molecules to recipient cells, thereby influencing their behavior and promoting bone regeneration.

**Figure 14:**
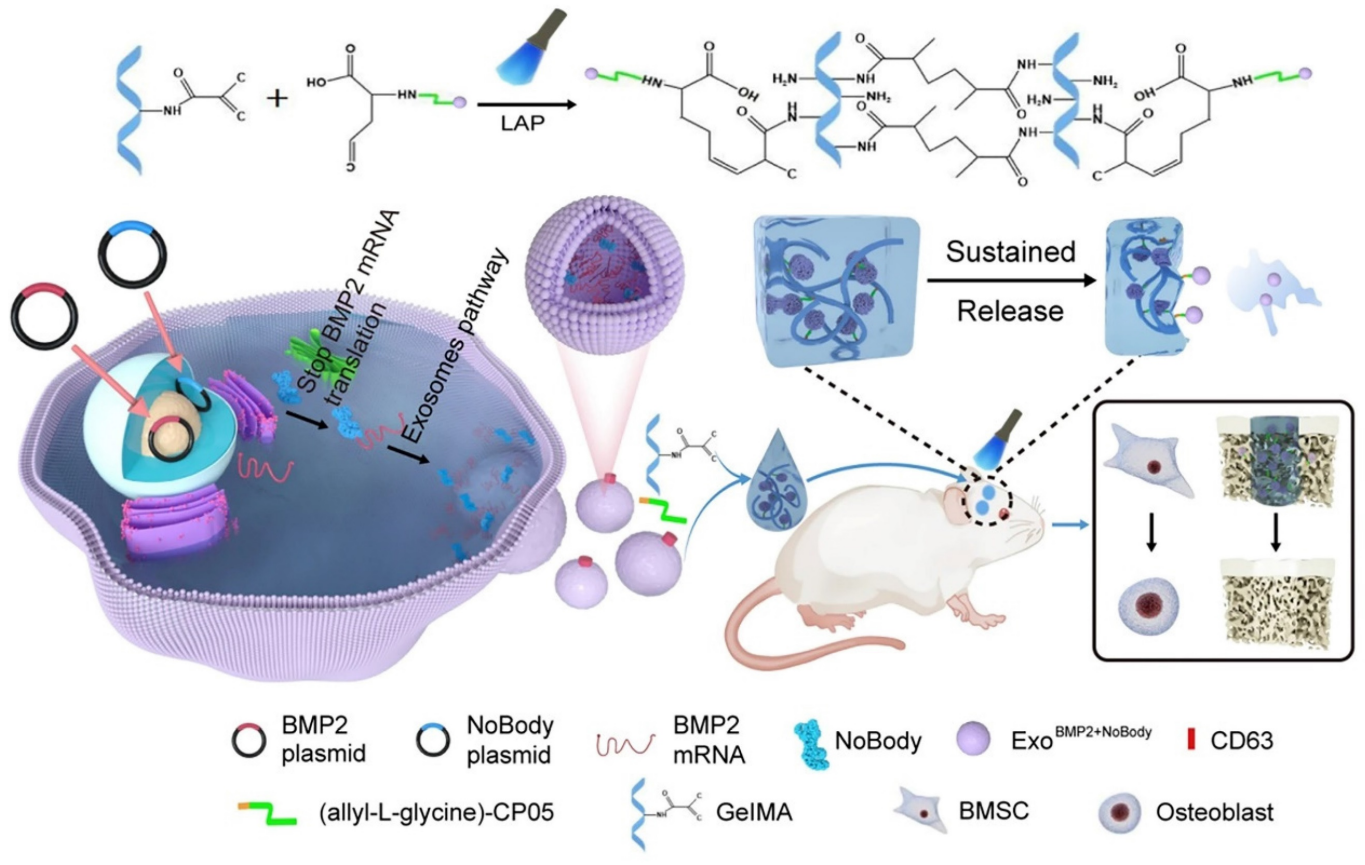
A schematic representation of the preparation process for Exo^BMP2+NoBody^-loaded GelMA and investigation of its impact on bone regeneration. Reproduced with permission from the ref. 151, Copyright 2023, Springer Nature (This work is licensed under CC BY 4.0. To view a copy of this license, visit http://creativecommons.org/licenses/by/4.0/).

Yang et al. 151 used genetic engineering to prepare exosomes that contained increased amounts of Bmp2 mRNA. This was achieved by cotransfecting synthetic NoBody and Bmp2 plasmids into 293T cells. The phage peptide CP05, which was modified with allyl-L-glycine, could capture exosomes originating from various sources by specifically binding to the exosome surface protein CD63. This study used GelMA as a substrate to covalently attach the allyl-L-glycine-modified CP05 peptide, thereby forming GelMA-CP05. The engineered exosomes were then used to investigate the osteogenic potential of stem cells in vitro and in vivo, focusing on achieving sustained release of the engineered exosomes. The findings of this study demonstrated that when the expression of target mRNAs was suppressed within the host cell, exosomes that were rich in the target mRNAs accumulated. The exosomes could be transported to the target cells, where they produced therapeutic proteins to treat diseases (Figure 14). Using this methodology, they developed the BMP2 and NoBody plasmids and effectively obtained modified exosomes, which exhibited enhanced levels of Bmp2 mRNA. The exosomes significantly influenced bone regeneration. The exosomes that were manipulated could bind to the hydrogel scaffold via a modified CP05. The successful integration of these components synergistically enhanced the continuous release of exosomes and facilitated osteogenesis in critical bone defects. This study presents a new and innovative approach to bone regeneration therapy.

Xu et al. 152 prepared an innovative scaffold created by electrospinning copper-based MOF (copper 1,4-benzenedicarboxylate (CuBDC)) with poly(lactic acid-co-glycolic acid) (PLGA) and immobilizing exosomes derived from human bone mesenchymal stem cells (hBMSC-Exos) onto fibrous polymer meshes. The exosome-laden scaffold with a unique nanostructure exhibited dual cooperative controllable release of bioactive copper ions and exosomes to promote osteogenesis and angiogenesis, thereby exerting therapeutic effects and enabling cell-free bone regeneration. The composite stent significantly increased the expression of osteogenic-related proteins (ALP, Runx2, Ocn) and VEGF in hBMSCs and promoted the migration and tube formation of human umbilical vein endothelial cells (HUVECs) in vitro. The scaffold's biocompatibility and osteogenic-angiogenic coupling effects were investigated in vitro, and bone repair was evaluated in vivo using a well-established bone defect model. This rational engineering strategy could effectively achieve vascularized osteogenesis while providing new insights into the design of cell-free fibrous scaffolds.

**Figure 15:**
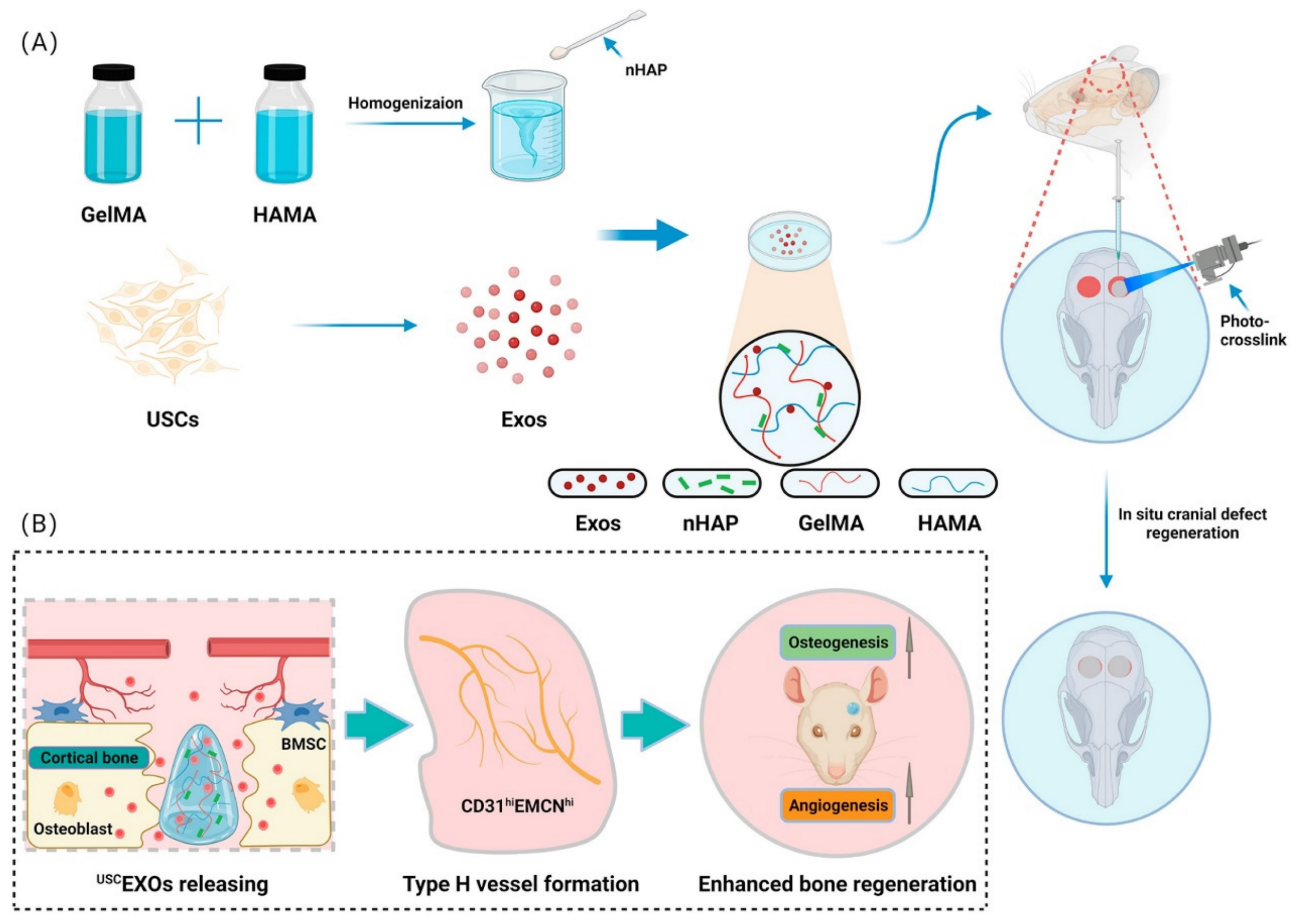
Novel photo-crosslinked hydrogels combined with human urine-derived stem cell exosomes have substantial merits in osteogenic repair. (A) Procedures for preparation of composite hydrogels. (B) Novel photo crosslinked hydrogels with instantaneous in situ seamless adhesion adapted to various bone defect morphologies and retaining human urine-derived stem cell exosome function to initiate osteogenesis and angiogenesis for cranial bone regeneration in rats. Reproduced with permission from the ref. 153, Copyright 2023, Elsevier (This work is licensed under CC BY-NC-ND 4.0. To view a copy of this license, visit http://creativecommons.org/licenses/by-nc-nd/4.0/).

Lu et al. 153 developed a bioscaffold hydrogel coated with growth factors that could be released under control conditions. They used gelatin methacrylate (GelMA) and hyaluronic acid methacrylate (HAMA) to create a composite hydrogel and then added nanohydroxyapatite (nHAP) to improve its mechanical properties. The USCEXOs (exosomes derived from human urine-derived stem cells) were encapsulated and systematically characterized to evaluate their physical properties, release rate, and osteogenic and angiogenic capabilities in vitro. The GelMA-HAMA/nHAP composite hydrogel exhibited excellent controlled release performance and suitable mechanical properties. In vitro studies demonstrated that the USCEXO/GelMA-HAMA/nHAP composite hydrogel could stimulate osteogenesis in bone marrow mesenchymal stem cells (BMSCs) and angiogenesis in endothelial progenitor cells (EPCs). Moreover, the in vivo results confirmed that this composite hydrogel could significantly promote cranial bone defect repair in a rat model. In addition, they discovered that the USCEXO/GelMA-HAMA/nHAP composite hydrogel could stimulate the formation of H-type blood vessels in the bone regeneration area, thereby enhancing the therapeutic effect. Their study provided a comprehensive understanding of the design of biocompatible and controllable GelMA-based hydrogels and a viable therapeutic strategy for critical bone regeneration (Figure 15).

Recent advances indicate that negotiation has significant potential for achieving multisystem modulations in bone tissue engineering (BTE). SC Exos are a viable therapy for peripheral nerve damage, and evidence suggests that SC-derived Exos have immunoregulatory and proangiogenic potential. As a result, they are great candidates for cell-free therapy to achieve neuralization in bone replacements. Hao et al.^154^ created a gelatin methacryloyl hydrogel (GelMA) to encapsulate SC Exos to maximize the use of SC Exos in vivo, and the long-term and sustained release of SC-exos in vivo significantly improved bone regeneration by encouraging innervation, immune regulation, vascularization, and osteogenesis. Furthermore, in vitro studies revealed that this system strongly enhanced M2 polarization in macrophages, tube formation by HUVECs, and osteogenic differentiation in BMSCs, and upregulation of the TGF-1/SMAD2/3 signaling pathway increased BMSC osteogenesis.

**Figure 16:**
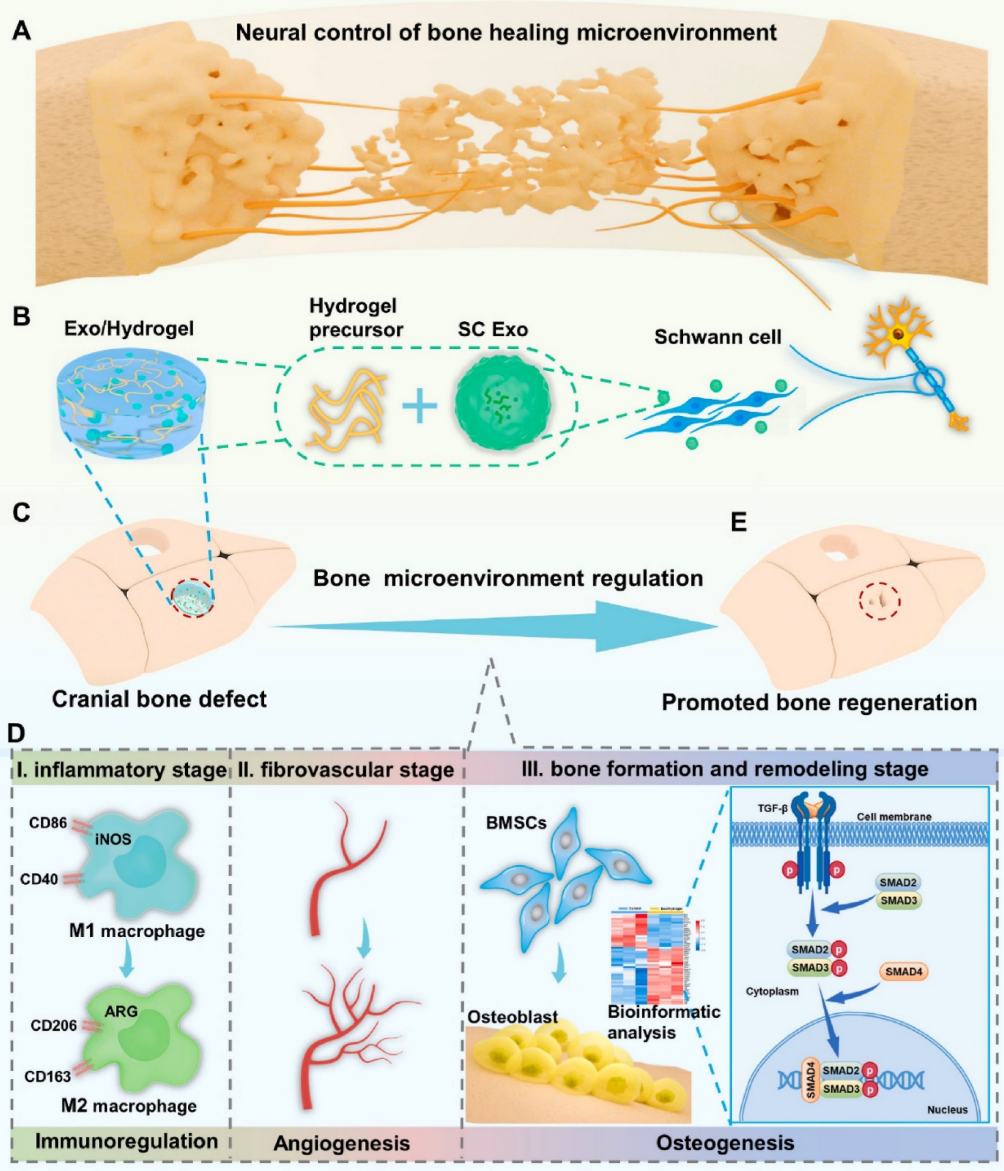
A diagram of neural engineering SC Exo for bone regeneration. (A) Nerves have a profound impact on bone regeneration by directing the bone microenvironment. (B) SC Exo isolation and Exo/Hydrogel fabrication. (C-E) Exo/Hydrogel application on cranial bone deformities. Exo/Hydrogel aided bone regeneration by synchronizing immunomodulation, angiogenesis, and osteogenesis during the inflammatory, fibrovascular, bone formation, and remodeling stages. Exo/hydrogel specifically triggered the TGF-signaling pathway to promote BMSC osteogenesis. Reproduced with permission from the ref. 154, Copyright 2023, Elsevier (This work is licensed under CC BY-NC-ND 4.0. To view a copy of this license, visit http://creativecommons.org/licenses/by-nc-nd/4.0/).

In conclusion, a novel cell-free and manufactured SC Exo neuroengineering system was successfully designed to stimulate bone regeneration by controlling the complete bone healing milieu; this strategy elicited a paradigm shift in bone regeneration and its relationship to the complete organ system, emphasizing a more comprehensive and integrated approach rather than focusing on the effects of components alone (Figure 16). Concurrent modulation of the immunological, vascular, and skeletal systems with SC Exos provides a simple yet innovative and beneficial cell-free method that can reveal new therapeutic insights and pathways for bone regeneration.

**Figure 17:**
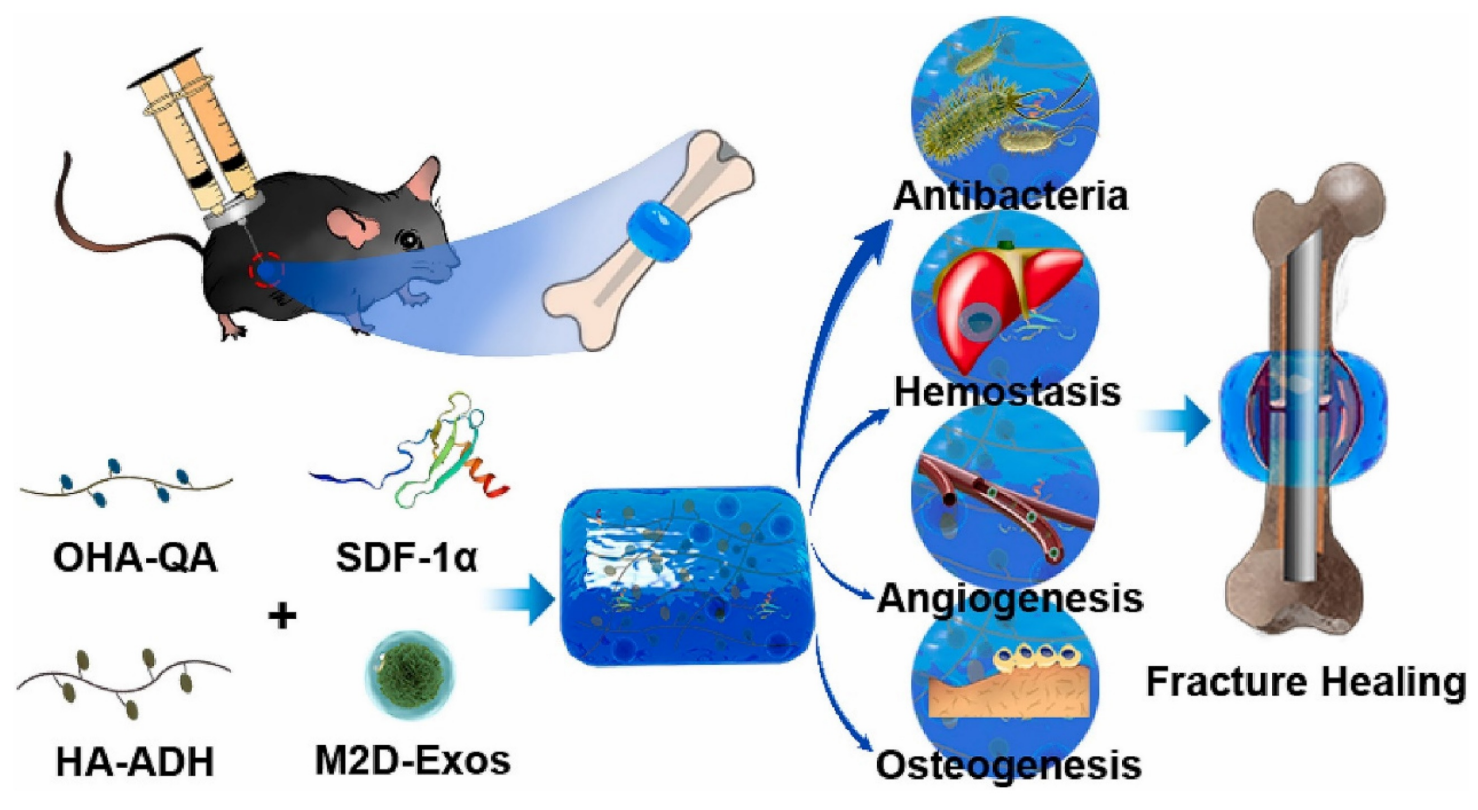
The processes underlie the potential of HA@SDF-1/M2D-Exos hydrogel to expedite fracture healing. A mixed injection technique can build the HA@SDF-1/M2D-Exos hydrogel in situ. Because of the hydrazone bond formation between the HA-ADH and OHA-QA crosslinking, the hydrogel formed quickly, while the positively charged quaternary ammonium groups of the hydrogel created a long-term antibacterial and hemostasis environment. The HA@SDF-1/M2D-Exos hydrogel increased osteogenesis and angiogenesis in vivo and in vitro by releasing SDF-1 and M2D-Exos synchronously and sustainably. Reproduced with permission from the ref. 155, Copyright 2023, Elsevier (This work is licensed under CC BY-NC-ND 4.0. To view a copy of this license, visit http://creativecommons.org/licenses/by-nc-nd/4.0/).

Fracture repair is divided into three stages: inflammation, repair, and remodeling. Significant progress has been made in bone regeneration, including the development of techniques to balance M1/M2 macrophage populations and promote osteogenesis and angiogenesis. However, these advancements have focused on one of the latter two phases while disregarding the inflammatory phase, which occurs during cell recruitment. Chen et al. 155 mixed stromal cell-derived factor-1 (SDF-1) and M2 macrophage-derived exosomes (M2D-Exos) with a hyaluronic acid (HA)-based hydrogel precursor solution and created an injectable, self-healing, sticky HA@SDF-1/M2D-Exo hydrogel by Schiff base interactions between adipic acid dihydrazide-modified HA (HA-ADH) and oxidized HA-quaternary ammonium (OHA-QA). The composite hydrogels showed efficient self-healing properties, outstanding biocompatibility, and hemostatic capabilities, and the 4% HA hydrogels exhibited excellent antibacterial activity against gram-negative *E. coli*, gram-positive *S. aureus*, and Methicillin-resistant *Staphylococcus aureus* (MRSA), ultimately contributing to improved fracture healing. The HA@SDF-1/M2D-Exo hydrogel enhanced the proliferation and migration of human bone marrow mesenchymal stem cells (HMSCs) and human umbilical vein endothelial cells (HUVECs), and the controlled SDF-1 release enhanced HMSC and HUVEC migration, whereas M2D-Exos increased cell proliferation, HMSC mineral deposition, and HUVEC tube formation, thus promoting osteogenesis and angiogenesis in vivo and in vitro (Figure 17). The overall design was compatible with the natural healing process of fractures, allowing for faster fracture healing while reducing infection. Overall, the HA@SDF-1/M2D-Exo hydrogel was compatible with the normal healing process of fractures and offered a new modality for accelerating bone regeneration by coupling osteogenesis, angiogenesis, and infection resistance at all phases.

While the potential of exosomes for bone regeneration is promising, additional research is needed to fully understand their mechanisms of action, optimize their therapeutic potential, and ensure their safety and efficacy. Clinical trials are underway to evaluate the use of exosomes in bone regeneration, and their use in regenerative medicine holds great potential for the future.

## 5. Conclusions and future perspectives

Exosomes have shown tremendous potential in tissue regeneration as natural intercellular communication vehicles. Because of their inherent ability to transport proteins, lipids, and nucleic acids, they are excellent candidates for therapeutic use. Because of their ability to induce cell proliferation, migration, and angiogenesis, exosomes produced from stem cells have emerged as promising new tools for tissue regeneration. Several studies have shown that modified exosomes loaded with specific miRNAs, proteins, or medicines have greater regeneration capacity than native exosomes. Engineered exosomes have demonstrated promising outcomes in preclinical investigations for bone, cartilage, skin, cardiac, neuronal, and other tissue regeneration. These findings suggest that these materials have potential for future clinical applications. Exogenous payloads can be loaded into exosomes through electroporation, transfection, fusion, scaffolding, or other methods. Exosome stability and targeting can benefit from further optimization of loading strategies. Exosomes can be coupled with biomaterial scaffolds or hydrogels to develop innovative tissue engineering structures.

Various preclinical investigations have shown that engineered exosomes have a favorable safety profile. These materials outperform manufactured nanoparticles due to their biocompatibility, minimal immunogenicity, and capacity to traverse biological barriers. One of the most significant advantages of modified exosomes is their ability to carry specific cargo. Exosomes can be guided toward particular cell types or tissues by modifying surface proteins or ligands, thereby boosting their regenerative potential. Engineered exosomes can be loaded with a variety of therapeutic agents, such as growth factors, miRNAs, and medicines, allowing for multiple approaches to tissue regeneration. The preliminary results of clinical trials have demonstrated the promise of exosome-based therapeutics for enhancing tissue repair and regeneration in illnesses such as myocardial infarction, liver disorders, and skin wounds.

Regulatory guidelines for exosome-based therapies are still evolving, and future research should focus on developing scalable and reproducible methods for clinical-grade exosome production; the standardization of exosome isolation, purification, and engineering techniques is one of the primary challenges in the field. Advances in exosome isolation, characterization, and engineering will allow for more sophisticated exosome designs with improved targeting and cargo loading. To create clinical grade exosomes, large-scale GMP production and consistent quality controls are needed.

The therapeutic effects of exosomes, as well as their modes of action, should be investigated further in the future. Precision medicine breakthroughs can lead to the development of patient-specific exosome medicines customized to individual needs and genetic backgrounds. Exosomes that have been engineered can be used with other therapeutic methods, such as stem cell therapy, to improve regeneration. Like any new treatment method, engineered exosomes must navigate a complex regulatory framework.

To determine the best exosome doses and treatment regimens, preclinical models should better mimic human physiology. The safety and efficacy of exosomes for specific treatments uses will be evaluated in early-phase clinical trials. The cost of creating and administering exosome-based medicines will also be a significant determinant of their widespread adoption. Efforts should be made to make these therapies economically viable for a broader range of people.

Finally, modified exosomes have enormous potential for tissue regeneration, providing a natural, adaptable, and targeted approach to therapy. Exosomes can be generated and used as a first-line regenerative therapy for injuries, degenerative disorders, and congenital impairments. Future studies could change regenerative medicine in the future if research and collaboration occur across fields.

## Figures and Tables

**Figure 1:**
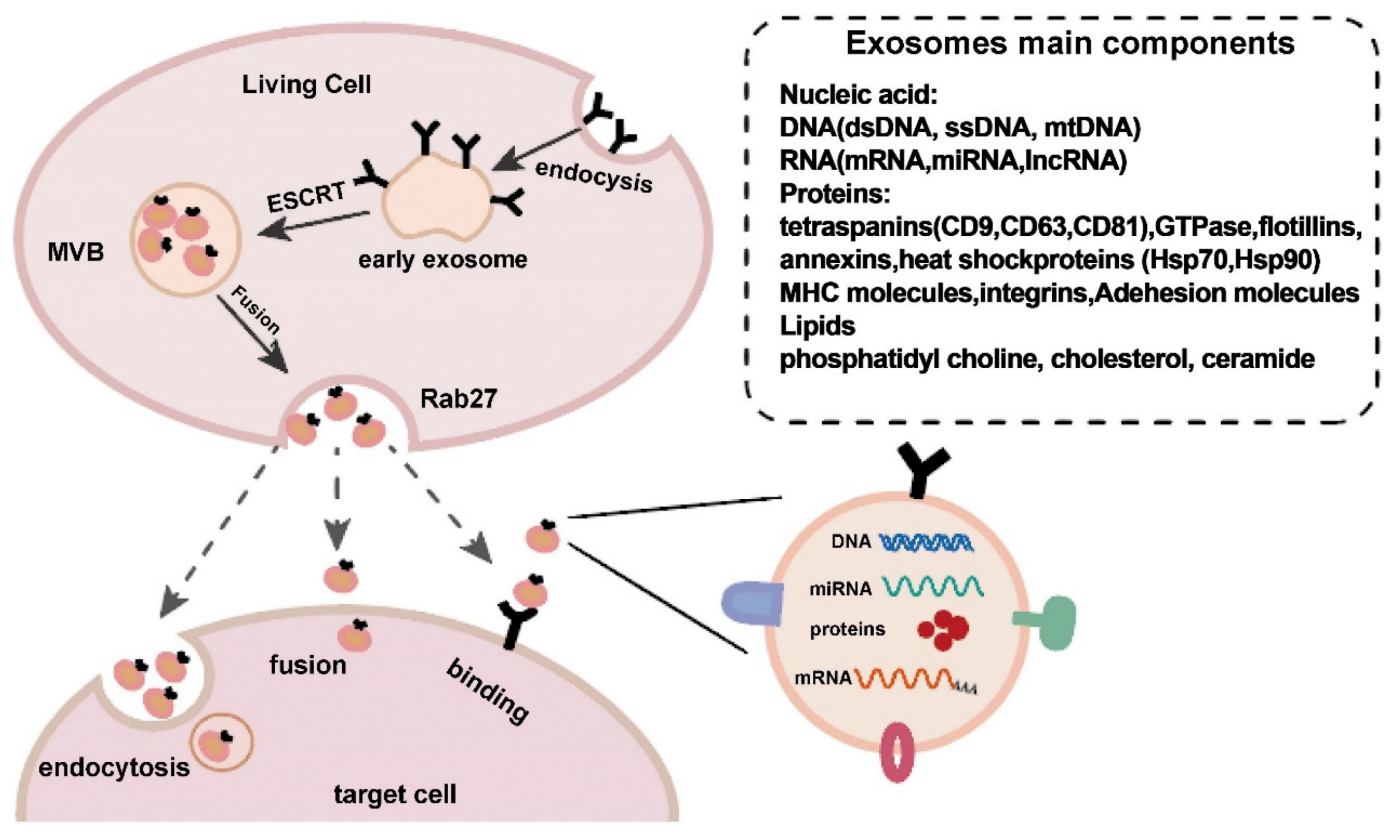
Exosome production and release. Exosome creation and release is a highly controlled process that begins with early endosomes in the membrane and progresses to multivesicular bodies (MVBs), which fuse with the cell membrane and are released into the extracellular space under the control of Rab27. Exosomes reach target cells by three mechanisms: fusion, endocytosis, and protein-receptor interactions. Reproduced with permission from the ref. 2, Copyright 2021, Elsevier (This work is licensed under CC BY-NC-ND 4.0. To view a copy of this license, visit http://creativecommons.org/licenses/by-nc-nd/4.0/).

**Figure 2:**
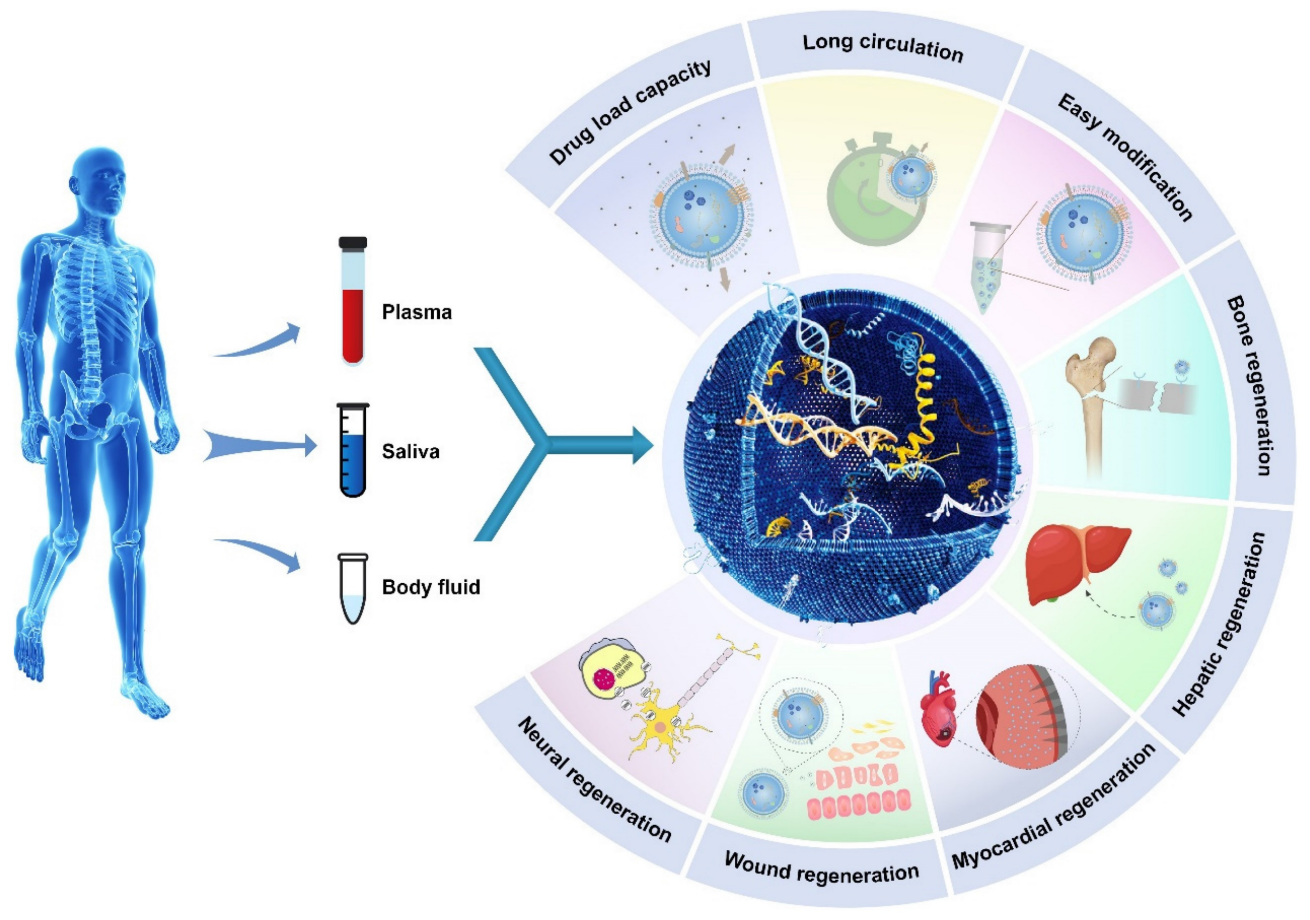
Illustrative schematic of exosomes' properties, origin, and applications in tissue engineering. The following section will discuss the role of exosomes in promoting tissue regeneration and repair.

**Table 1:** The proposed classification of exosome isolation methods is based on their primary principles. The advantages and disadvantages of each method are highlighted. The feasibility of potential applications based on isolation methods is also described.

Method	Type	Advantages	Disadvantages	Applications			
				Nucleic acid quantification and sequencing	Biomarker screening	Protein quantification and identification	Drug delivery systems
Ultracentrifugation	Physical	• Easy procedures • Maintaining physical and chemical properties • Higher separation efficiency • preservation integrity	• Low purity • Equipment needed for specialized tasks • Time consuming • Unrelated proteins can contaminate exosomes.	Yes	Yes	N/A	Yes
Ultrafiltration/Nano-filtration	Physical	• Quick procedures • Material inexpensive • Excellent protein and RNA yield • Commercial kits available	• Purity low • Deformation and extrusion of exosomes • Risk of contamination from non-exosomal humoral peptides • Filter determines exosome recovery	Yes	Yes	N/A	N/A
Precipitation	Physicochemical	• Highly repeatable • Higher vesicle and RNA yields • Only lab bench equipment needed	• Low purity • Exosome aggregation by polymer impeding exosome research via -omics assays due to positively charged molecules and pellet contamination.	Yes	Yes	N/A	N/A
Size-exclusion chromatography	Physicochemical	• High yields of pure exosomes • Preservation of physicochemical properties.	• Processing times: medium to high • Selected operation buffers cause exosome degradation.	Yes	Yes	Yes	Yes
Microfluidics	Physical	• Miniaturization • Device functionalization • Short analysis times	• Low reproducibility • Poor exosome yields • Aggregating exosomes	Yes	Yes	Yes	Yes
Immunoaffinity	Chemical	• Highly specific • Fine purity • Possible scaling	• Immunoaffinity capture, requiring large amounts of antibody-conjugated beads, is an expensive method for isolating exosomes from large samples. • Low exosomal and RNA yields	Yes	N/A	Yes	N/A

**Table 2:** Principle, advantages, and disadvantages of producing exosome-laden biomaterials

	Principle	Advantages	Disadvantages	Applications
Exosomes grafted onto the surface of biomaterials	• Physical Adsorption • Bio-affinity Binding • Covalent Bonding • Layer-by-Layer Assembly • In Situ Coating	• Localized Delivery • Sustained Release • Protection from Clearance • Immunomodulation • Versatility	• Limited Loading Capacity • Variable Loading Efficiency • Immunogenic Concerns	• Tissue Engineering Scaffolds • Bone Implants • Wound Healing Patches • Cardiac Patches
Exosomes encapsulated in the biomaterials	• Hydrogel Encapsulation • Electro-spraying • Layer-by-Layer Assembly • Microspheres/Microcapsules	• Protection • Sustained Release • Cargo Versatility • Scalability	• Loading Efficiency • Immunogenicity • Manufacturing Limitations • Characterization Complexities	• Tissue engineering • Immunotherapy • Chronic wound healing • Cardiac repair • Neural regeneration • Osteogenic grafts • Metabolic therapy
Exosomes integrated with nanoparticles	• Incubation • Electroporation • Sonication • Lipid Fusion • Chemical Conjugation • Layer-by-Layer Assembly • Microfluidic Manipulation	• Multimodal Loading • Targeting • Stimulus-Response • Enhanced Uptake • Protection • Scalability	• Loading Variability • Cargo Interference • Toxicity Concerns • Immunogenic Triggering	• Cancer Therapy • Vaccine Delivery • Neurotherapies • Tissue Engineering • Bioimaging • Biosensing
